# The Interplay of Diet Quality and Alzheimer’s Disease Genetic Risk Score in Relation to Cognitive Performance Among Urban African Americans

**DOI:** 10.3390/nu11092181

**Published:** 2019-09-11

**Authors:** Sharmin Hossain, May A. Beydoun, Marie F Kuczmarski, Salman Tajuddin, Michele K Evans, Alan B Zonderman

**Affiliations:** 1 Laboratory of Epidemiology and Population Sciences, National Institute on Aging Intramural Research Program (IRP), National Institutes of Health, Baltimore, MD 21224, USA; 2 Department of Behavioral Health and Nutrition, University of Delaware, Newark, DE 19716, USA; 3 Biomedical Research Center, National Institute on Aging (NIA), 251 Bayview Blvd. Baltimore, MD 21224, USA

**Keywords:** genetic risk score, diet quality, Alzheimer’s Diseases, cognitive function, working memory, African American, single nucleotide polymorphism, health disparities

## Abstract

We examined the interactive associations of poor diet quality and Alzheimer’s Disease (AD) genetic risk with cognitive performance among 304 African American adults (mean age~57 years) from the Healthy Aging in Neighborhoods of Diversity across the Life Span (HANDLS) study. In this cross-sectional study, selected participants had complete predictors and covariate data with 13 cognitive test scores as outcomes. Healthy Eating Index-2010 (HEI-2010), Dietary Approaches to Stop Hypertension (DASH), and mean adequacy ratio (MAR) were measured. A genetic risk score for AD in HANDLS (hAlzScore) was computed from 12 selected single nucleotide polymorphisms (SNPs). Our key hypotheses were tested using linear regression models. The hAlzScore was directly associated with poor performance in verbal memory (−0.4 ± 0.2, 0.01) and immediate visual memory (0.4 ± 0.2, 0.03) measured in seconds, in women only. The hAlzScore interacted synergistically with poorer diet quality to determine lower cognitive performance on a test of verbal fluency. Among numerous SNP × diet quality interactions for models of cognitive performance as outcomes, only one passed correction for multiple testing, namely verbal fluency. Our results suggest that improved diet quality can potentially modify performance on cognitive tests of verbal fluency among individuals with higher AD genetic risk.

## 1. Introduction

Mild cognitive impairment (MCI) is defined as greater than expected cognitive decline for an individual’s age and education level (<High School = 0, High School = 1 and >High School = 2) without marked interference in daily activities [1]. Poor lifestyle factors like reduced diet quality, often tightly linked with lower socioeconomic status [2], are associated with poorer cognitive performance among older adults [3,4], whereas high quality diets are protective and restorative of cognitive function [5,6,7,8]. The Mediterranean diet—rich in fruits, vegetables, nuts/beans/seeds, and heart healthy fats—is an example of a high quality diet, which decelerates cognitive decline [9] and lowers the risk of associated chronic neurological diseases [8,10,11] including Alzheimer’s and Parkinson’s diseases. The DASH (Dietary Approaches to Stop Hypertension) diet is very similar to a typical Mediterranean diet and has been widely used and recommended in the US, especially by the American Heart Association (AHA).

Single nutrients like omega-3 fatty acids and B-vitamins [12] have been reportedly essential for optimal brain functions. Studies have examined AD risk by Apolipoprotein E (APOE) ε4 carrier status in relation to some of these nutrients [13,14,15,16], though the evidence remains limited. Alternatively, diets characterized by being high in salt, alcohol, unsaturated fat and low in dietary fiber [17] are linked with poor cerebral blood supply, increased inflammation, and subsequent neurological impairment [18]. However, a composite measure of diet quality [19] is more potent than single nutrient analyses for cognition and related neurological outcomes. 

Although the evidence on diet quality and cognitive function is largely convincing, the role of genetic risk factors in influencing this association remains unknown. The primary aim of the current study was to examine associations of genetic risk scores with cognitive performance, while testing genetic risk by diet quality interactions in a subset of African American participants (cognitively intact and without diagnosis of MCI or dementia) from the Healthy Aging in Neighborhoods of Diversity across the Life Span (HANDLS) study. A second aim was to further examine genetic risk × diet quality interactions in relation to cognitive function, separately among men and women. To date, this is the first investigation among African Americans of cognitive performance in relation to both genetic scores for Alzheimer’s risk and diet quality.

## 2. Materials and Methods 

### 2.1. Data Source

The HANDLS study [20] investigates health disparities in an area probability sample of working age African American and white adults in Baltimore City, Maryland. The initial cohort consisted of 3720 men and women who were 30–64 years old. The present study examines data from the initial wave conducted from 2004–2009. In the first wave, data were collected in two phases. In the first phase, participants were recruited from their homes where they were administered a household questionnaire and the first of two 24 h dietary recall interviews. In the second phase, participants were examined on Mobile Research Vehicles where they were administered neuropsychological tests, physical examinations, medical histories, and the second of two 24 h dietary recall interviews.

### 2.2. Participants

**Figure 1:**
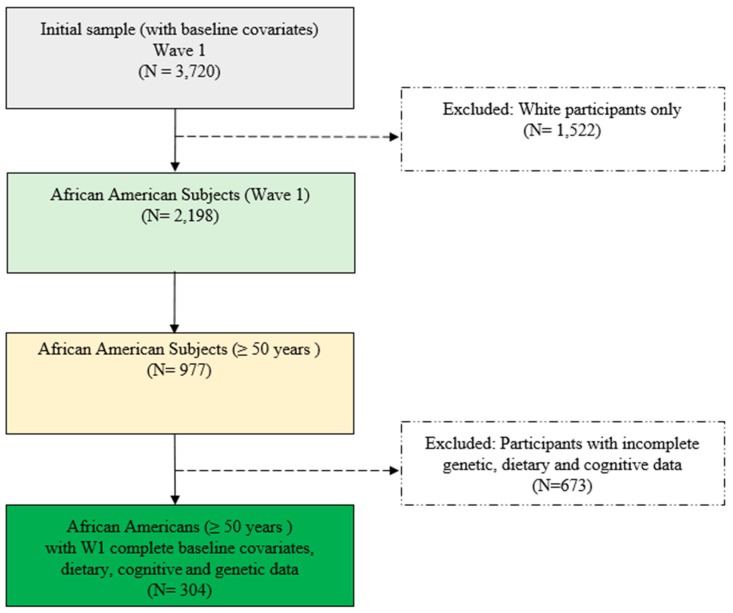
Chart of subject section.

Of the initial 3720 participants, 2,198 were African Americans (59.0%). Of those, we selected participants ≥50 years of age (*N* = 977; 44.4%) who had complete dietary data (*N* = 554; 55.6%). The sample size was further constrained by the availability of complete genetic information (*N* = 480; 49%) and cognitive tests (*N* = 398; 40.7%) from the initial sub-sample of 977 participants. Exclusion criteria, which included incomplete cognitive tests, partial genetic, dietary and covariate data, yielded a final sample of 304 participants (32.3%) for analysis (Figure 1).

### 2.3. Dietary Methods and Quality 

#### 2.3.1. Method

All 24-hour dietary recalls were collected using the United States Department of Agriculture (USDA) computerized Automated Multiple-Pass Method (AMPM) [21]. The AMPM involves five steps designed to provide cues and prompts thorough recall for all foods and drinks consumed throughout the previous day [22]. These steps include (1) quick list of all foods consumed the previous day; (2) a forgotten foods list which includes probes for commonly forgotten foods; (3) probes to determine the time a food was consumed as well as at which meal; (4) detailed questions including amounts of foods consumed, additions to foods, and where food was obtained; (5) a final review probe to collect any food not previously remembered. Trained dietary interviewers conducted both 24-hour dietary recalls approximately 4–10 days apart. Measurement aids, including an illustrated food model booklet, measuring cups, spoons, and ruler assisted participants in estimating accurate quantities of foods and beverages consumed. Each recall was coded using the USDA Survey Net data processing system to match the foods with codes in the Food and Nutrient Database for Dietary Studies [23]. Of the 3720 participants examined in the baseline study, 2177 individuals completed two 24-hour dietary recalls.

#### 2.3.2. Healthy Eating Index 2010 (HEI2010)

Food-based diet quality was also evaluated with the HEI-2010. The National Cancer Institute’s Applied Research website provided the basic steps for calculating the HEI-2010 component and total scores and statistical codes for 24-h dietary recalls [24]. A detailed description of the procedure used for this study is available on the HANDLS website [25]. Component and total HEI-2010 scores were calculated for each recall day and were averaged to obtain the mean for both days combined.

#### 2.3.3. Dietary Approaches to Stop Hypertension (DASH)

The score for DASH diet adherence, based on 9 nutrients, was determined for each participant using the formula reported by Mellen et al. [26]. The nine target nutrients were total fat, saturated fat, protein, fiber, cholesterol, calcium, magnesium, sodium and potassium. Micronutrient goals were expressed per 1000 kcal. The total DASH score was generated by the sum of all nutrient targets met. If the participant achieved the DASH target for a nutrient, a value of 1 was assigned, and if the intermediate target for a nutrient was achieved, a value of 0.5 was assigned. A value of zero was assigned if neither target was met. The maximum DASH score was 9; individuals meeting approximately half of the DASH targets (DASH score = 4.5) were considered DASH adherent [26].

#### 2.3.4. Mean Adequacy Ratio (MAR)

Diet quality was also assessed using Nutrient Adequacy Ratio (NAR) and Mean Adequacy Ratio (MAR) scores [27,28]. The NAR score was determined by taking each participant’s daily intake of a nutrient divided by the Recommended Dietary Allowance (RDA) for that nutrient. NAR scores were determined for 17 micronutrients: vitamins A, C, D, E, B6, B12, folate, iron, thiamin, riboflavin, niacin, copper, zinc, calcium, magnesium, phosphorus, and selenium. The RDA was adjusted for participants’ ages and sexes and vitamin C was adjusted for smokers [29]. The NAR score was converted into a percent with values exceeding 100 truncated to 100. MAR scores were calculated by averaging the NAR scores: MAR = (∑NAR scores)/17 [30]. NAR and MAR were calculated separately for each daily-intake and then averaged. MAR scores, based on food intakes only, were used as the nutrient-based diet quality variable.

#### 2.3.5. Cognitive Measures

A cognitive battery of tests was administered to participants consisting of: Mini-Mental State Examination (MMSE); California Verbal Learning test–List A (CVLT-List-A); California Verbal Learning Test–Free Recall Long Delay (FRLD); Benton Visual Retention Test (BVRT); Brief Test of Attention; Trailmaking Test A (Trails A); Trailmaking Test B (Trails B); Digits Span Forward Test; Digits Span Backward Test; Clock Command Test; Identical Pictures Test; Card rotation Test; and Verbal fluency Test. Details of these tests are available in Supplementary material 1. Except for BVRT and the Trailmaking Tests, better performance was measured by higher scores. For BVRT and Trailmaking Tests parts A and B, better performance on BVRT was measured by fewer errors; the Trailmaking Tests was measured by faster performance. 

#### 2.3.6. Covariates

Selected covariates consisted of sociodemographic variables, depressive symptoms, health behaviors, lifestyle factors, inflammatory and cardiovascular outcomes. They were selected based on reported significant correlations with diet quality or cognitive function from the literature. 

Socio-demographic characteristics included baseline age, sex, race, poverty status, and educational attainment. Age was measured in years and used as a continuous variable in models. Race was dichotomized by self-identification as African American or White, and only African Americans were selected for the current study. Poverty status was dichotomized using the US Census Bureau, below or above 125% of the poverty thresholds for 2004 [31] based on income, size of family and related children under age 18 years. Educational attainment was categorized as fewer years than high school (HS), HS graduation or GED, and post-HS education. Lifestyle and health-related covariates included measured body mass index (BMI, kg/m^2^), self-reported opiate, marijuana, or cocaine use (“current” vs. “never or former”), smoking status (“current” vs. “never or former”), and the Wide Range Achievement Test (WRAT) scores to measure literacy. Depressive symptomatology was assessed with the Center for Epidemiologic Studies Depression Scale (CES-D). Overall dietary quality was assessed based on two self-reported 24-h recalls administered at baseline and reported as total score from the Healthy Eating Index (HEI-2010). Finally, self-reported history of several chronic diseases and medication history from the first visit in HANDLS, were used as other covariates. These are namely: diabetes, hypertension, dyslipidemia, cardiovascular disease (stroke, congestive heart failure, non-fatal myocardial infarction. or atrial fibrillation), inflammatory disease (multiple sclerosis, systemic lupus, gout, rheumatoid arthritis, psoriasis, thyroid disorders, and Crohn’s disease), and use of non-steroidal anti-inflammatory drugs (NSAIDs, prescription, and over the counter) over the past 2 weeks.

#### 2.3.7. Genetic Data 

1024. participants were successfully genotyped to 907763 single nucleotide polymorphisms (SNPs) at the equivalent of Illumina 1M array coverage. Sample exclusion criteria were (1) call rate <95%, (2) discordance between self-reported sex and sex estimated from X-chromosome heterogeneity, (3) cryptic relatedness, (4) discordance between self-reported African ancestry and (5) ancestry confirmed by genetic data. SNP exclusion criteria were (1) Hardy-Weinberg equilibrium p-value <10^−7^, (2) minor allele frequency <0.01, and (3) call rate <95%. Genotype quality control and data management was conducted using PLINKv1.06 (PMID: 17701901). Cryptic relatedness was estimated via pairwise identity by descent analyses in PLINK and confirmed using RELPAIR (PMID: 11032786). HANDLS participant genotypes were imputed using MACH/minimac version 2.0 (https://genome.sph.umich.edu/wiki/Minimac) based on combined haplotype data for the 1000 Genomes Populations project phase 3 version 5 multi-ethnic reference panel. 

#### 2.3.8. Genetic Risk Score Calculation

Previously reported genetic variants at various genetic loci implicated with phenotypes of Alzheimer’s Disease (AD) [32] were used for genetic risk score calculation (Table S1). Of the one hundred-thirty reported genetic variants, seventy-seven had valid SNP identifier. Seventy out of seventy-seven SNPs had imputed genotype data in the HANDLS study. After excluding two SNPs with poor imputation quality score (*R*^2^ < 0.30), there were 68 SNPs for the final analysis. The 68 SNPs were screened for significant associations with the MMSE total score from the published studies, since MMSE was the principal cognitive test used in the related literature. Only 12 [33,34,35,36,37,38,39,40] of the 68 SNPs showed significant MMSE association with baseline cognitive performance, across sex, age, race, and geographical location. HANDLS SNPs that were imputed had a quality score between 0.97 and 0.99. It is noteworthy that the majority of the 68 sorted SNPs were present in Whites, therefore increasing our inability to transfer more than 12 to African Americans. To calculate the risk score, the dosage alleles were identified first (based on the genotype data). These alleles were then cross-validated against SNPedia [41]. The risk alleles were identified using the AlzGene database. The total value for each SNP (from GWAS analysis) ranged between 0 and 2. If values between 0 and 0.4 were higher in frequency than 1.6 and 2.0, as percent (%) total, then the dosage allele was recognized as the minor allele and vice versa. After cross-validation and reordered directionality, the SNPs were labelled with corresponding gene and loci information, e.g. if the dosage allele for rs10503 was C then a renaming was rs10503_C. Next, categorical variables were created for each SNP using 0, 1 and 2 as the desired levels where 0 = values between 0 and <0.5, 1 = values between 0.5 and <1.5 and 2 = values between 1.5 and 2.0. Imputed SNPs were also categorized the same way. Finally, the sum of the renamed and labelled dosage alleles of these 12 SNPs were used for the calculation of the HANDLS Alzheimer’s disease genetic risk score (hAlzScore) as below: a) if the allele in question, e.g., C is a risk allele (i.e., it increases the risk of AD), then the dosage allele is kept the same. b) if the allele in question, e.g., C is a protective allele (i.e., it decreases the risk of AD), an inverse variable was created for each of the SNP; and c) if the minor allele is the risk allele, it was then processed the same way as (b). Table 1 presents the correlation matrix of the individual SNP by hAlzScore. The SNPs were located on *TF* (*n* = 1), CST3 (*n* = 1), *PSEN1* (*n* = 1), *PRNP* (*n* = 1), *IDE* (*n* = 1), *TFAM* (*n* = 1), *APOE* (*n* = 2), *ACE* (*n* = 2), *GAPDH* (*n* = 1) and *CHRNB2* (*n* = 1).

#### 2.3.9. Statistical Analyses

Analyses were performed by Stata SE Version 15.0 [42], consisting of several steps. First, we described selected sample characteristics by sex. Means of continuous measures were compared using independent samples *t*-test, while categorical covariate proportions were compared by sex using a χ^2^ test of independence. All variables were assessed for outliers and assumptions of normality. Second, we examined the association of (A) hAlzScore and (B) three diet quality measures (HEI-2010, DASH, and MAR scores) with cognitive performance by separate linear regressions of each cognitive test scores, in the total sample and stratifying by sex. All models were adjusted for demographic and health measures age, race, sex, poverty status, educational attainment, and lifestyle and health-related factors namely BMI, mean energy at baseline, CES-D score, and dichotomous current smoking status, current drug use, NSAIDS use, and diagnoses of diabetes, hypertension, high cholesterol, cardiovascular disease, and autoimmune conditions. Heterogeneity of main associations by sex were tested in separate models with 2-way interactions between hAlzScore/diet quality and sex. Third, in the total sample and separately by sex, diet quality and hAlzScore (and individual SNPs for hAlzScore) were examined to assess gene×diet interaction in relation to cognitive performance, while stratifying by sex. Three-way interactions between gene, diet, and sex were also assessed separately. Type I error was set at 0.05 for main effects and 0.10 for 2-way or 3-way interaction terms. We adjusted for selection bias due to non-participation using 2-stage Heckman selection model by adding an inverse mills ratio to the main effects in the linear regression models [43,44].

At the first stage, a probit model was conducted with a sample selection variable (0 = not selected, 1 = selected) as the outcome and complete socio-demographic variables as the covariates. An inverse mills ratio was obtained as a transformation of the predicted probability to be selected, given the covariate distribution. At a second stage, this inverse-mills ratio which is usually has a mean close to zero, was entered into our final causal models, as done in previous studies [45]. All continuous covariates and inverse mills ratios were centered at their mean. Parameter estimates from regression models and test statistics are expressed as (β ± Standard Errors, *p*-value). A familywise Bonferroni correction for multiple testing was carried out taking into account multiplicity in diet quality and SNP measures, while assuming cognitive performance outcomes and SNP measures as distinctive substantive hypotheses [42]. Thus, type I error for diet quality main effects was reduced to 0.017 and that of SNP main effects to 0.05/12 = 0.004. For 2-way interaction terms, type I error of hAlzScore × diet quality was reduced to 0.10/3 = 0.033 and that of SNP × diet quality interaction terms to 0.10/36 = 0.0028. Finally, to illustrate some of the key findings, predictive margins from multiple linear regressions were presented to highlight interactive relationships between the AD genetic risk score and diet quality in determining cognitive performance. 

## 3. Results

### 3.1. Sample Characteristics

Upon sample selection, 304 participants had complete and valid data on the MMSE, main predictors and covariates, whose mean age was ~57 years for both genders combined (53.6% women, 46.3% men) (Table 2). Around 44% of the sample consisted of participants with household incomes <125% of the poverty line. Mean BMI was 30.5 ± 0.4 kg.m^−2^ with women exceeding the criterion for obesity (32.6 ± 0.7 kg.m^−2^ ) and men meeting the criterion for overweight (28.0 ± 0.5 kg.m^−2^), (*p* < 0.001, *t*-test). In contrast, men reported a higher proportion of current illicit drug use (*p* = 0.007) and cigarette smoking (*p* = 0.001) compared to women. Despite no detectable differences in DASH and MAR by sex, mean HEI-2010 reflected a better overall dietary quality among women compared to men (*p* = 0.006). Self-reported chronic conditions such as hypertension (*p* = 0.04) and inflammatory conditions (*p* = 0.001) were also higher in women, along with marked differences in baseline cognitive performance by sex whereby women performed better on CVLT- List A (*p* < 0.001), CVLT- FRLD (*p* < 0.001), and Verbal fluency (*p* = 0.03), while men scored higher on the Card Rotation test (*p* < 0.001). 

A comparison between the excluded and analyzed participants (Table S5) was also performed. Differences were detected by education level (*p* = 0.001) and poverty status (*p* = 0.02), whereby the less educated and lower income groups were more likely to participate, with no differences found by sex, race or age. 

Overall, CVLT-FRLD (−0.4 ± 0.2, 0.01) and BVRT (0.4 ± 0.2, 0.03) were associated with hAlzScore with the same associations found mainly among women [CVLT-FRLD (−0.5 ± 0.2, 0.04) and BVRT (0.7 ± 0.3, 0.007)] (Table 3). 

We also investigated the relationship between individual SNPs comprising hAlzScore and the cognitive tests (Table S2), stratified by sex. All but one cognitive test (CVLT-List A) showed some degree of association with select SNPs and the results varied when stratified by sex. 

### 3.2. Cognitive Tests and Their Association with Diet Quality Indices

Diet quality was also examined in relation to cognitive test performance, stratifying by sex and adjusting for multiple covariates (Table 4). None of the associations were statistically significant (*p* > 0.017) after correcting for multiple testing. 

### 3.3. hAlzScore Interaction with Diet Quality Scores in Relation to Cognitive Tests 

Table 5. displays 2-way interaction terms (*p* < 0.10) between hAlzScore and diet quality indices in multiple linear regression models of cognitive performance. Taking a threshold of *p* < 0.033 (testing for multiple corrections; *p*-value 0.10/3 = 0.033 for three dietary quality indices), two associations were statistically significant. HEI-2010 had a potential protective effect on a test of verbal fluency (AF) at higher levels of the hAlzScore, denoting a synergistic interaction between poor diet quality and AD genetic risk in relation to verbal fluency domain of cognition. This interaction was specific to women *(p* = 0.02). The full results including main effects of diet and hAlzScore are presented in Table S3a.

### 3.4. SNP Interaction with Diet Quality Scores in Relation to Cognitive Tests 

**Figure 2:**
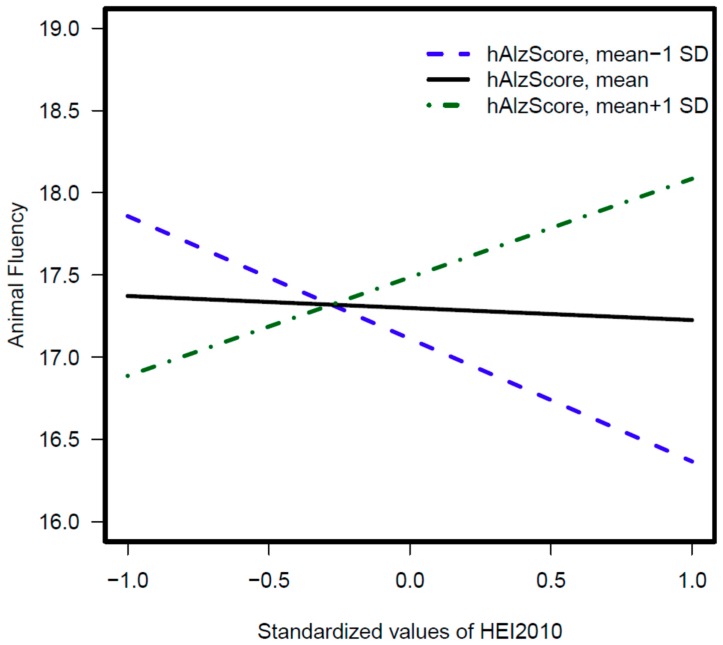
Predictive margins for animal fluency test scores by standardized z-scores of hAlzScore and HEI- 2010: Linear regression with 2- way interaction between gene and diet quality. Abbreviations: hAlzScore = HANDLS Alzheimer’s Disease genetic risk score; HEI-2010 = Healthy Eating Index, 2010 version.

We also conducted OLS regression analyses, whereby a 2-way interaction between each individual SNP and diet quality index was included in addition to their main effects and those of potentially confounding covariates. Outcomes were the 13 cognitive test scores measured at baseline. Models were stratified by sex and gender differences were tested using 3-way interaction. Results are displayed for each diet quality index in Tables S3a-c and described in Supplementary material 2. Standardized *z* -scores of hAlzScore interacted synergistically with those of poorer diet quality to determine lower cognitive performance on a test of verbal fluency. Models indicated that moving from a high quality (at mean + 2 SD) to a medium quality diet (at mean) can specifically predict poorer performance in the domain of verbal as illustrated in Figure 2. A similar interaction was observed when examining tertiles of hAlzScore and those of HEI-2010 in relation to the Verbal fluency test (data not shown). 

## 4. Discussion

To our knowledge, the present investigation is the first to examine potential interactive relationships of genetic risk for Alzheimer’s Disease and diet quality in a predominantly African American population, with multiple measures of cognitive function. We found that AD genetic risk was associated with measures of poorer performance on measures of verbal memory and visual memory, particularly among women. Although some individual SNPs were linked to cognitive performance (e.g., rs165932 (“T” allele) and Digits Span-Forward, total population), (*P* < 0.004)), upon correction for multiple testing, none of the dietary quality indices were linked to cognitive performance (*p* > 0.017). However, hAlzScore interacted synergistically with poorer diet quality to determine lower cognitive performance on a test of verbal fluency. Some SNP × diet quality interactions were also detected among men and women separately for tests of verbal fluency, executive function, and visuospatial ability, though with inconsistent directionality. Some cognitive domains are sensitive to behavioral factors such as diet, while others are determined by genetic risk, and a third group is determined by the interaction of genetic risk with behavioral factors. Our study is a first step to determine which domains are determined by synergism between genetic risk and poor diet. However, more studies are needed to uncover those specific domains

Of all the known risk factors of AD, age is the strongest followed by apolipoprotein E (APOE) gene variation. While the ε4 variant of APOE gene has been associated with increased AD risk, ε2 is associated with decreased AD risk according to a recent systematic review [46] on the risk factors associated with the onset and progression of AD. Using the AlzGene database, APOE ε2 was suggestive of a protective effect (OR = 0.62; 95% CI = 0.46, 0.85; I^2^ = 64%). There are two APOE ε2 SNPs (rs405509 and rs449647), used in the creation of hAlzScore, that are directly imputed in HANDLS. These SNPs were correlated with approximately 50% of the cognitive tests administered. MMSE scores were inversely related to rs449647 in women only, which is consistent with existing literature on AD risk. 

Despite the lack of support for our hypotheses about the potential benefit of diet quality on cognitive performance, we found some notable gene by diet quality interactions. These interactions suggested that diet quality is directly related to cognitive performance on a test of verbal fluency among individuals at higher risk for AD. The overall diet quality was quite low in our selected sub-sample, yet we detected several differences in cognitive performance that varied by sex. However, one recent study on healthy older adults showed there is more room to improve cognition by improving diet quality in low SES groups than their high SES counterparts [47].

Studies on diet quality and cognitive performance demonstrated that a diverse diet with good supply of macro and micronutrients [48], reduced alcohol intake [49] and increased physical activity [50,51] are helpful in attenuating mild cognitive impairment (MCI) [52] and progression to AD in older adults [53,54]. Preventing or delaying the onset of MCI can lead to a substantial improvement in quality of life. Growing evidence supports the protective role of diets rich in fish, heart-healthy oils, fresh fruits and vegetables in reducing risk for MCI [55,56,57] as well as early stages of dementia [58]. These studies show that higher adherence to a Mediterranean-type diet [59] as described previously, could be a reason behind slower cognitive decline, delayed development of dementia and reduced risk of progression from MCI to AD. Greater adherence to the DASH (Dietary Approaches to Stop Hypertension) diet, despite lower SES, could be beneficial in our population.

Although most of our findings are based on the composite risk score of the 12 selected SNPs, when we performed the same analyses individually, both APOE SNPs were highly correlated with more than one dietary index and in multiple cognitive tests when stratified by sex. This has potential implications when interpreting the results because we are working with a community-dwelling cohort of African Americans. We can posit that dietary interventions, in combination with genetic susceptibility markers for AD in a community cohort could have the potential for a better management of the outcome in the long run. This could lead to a greater emphasis on improving the diets of socioeconomically disadvantaged populations to improve or preserve cognitive function at young ages as part of the greater public health implications.

A decline in cognitive performance and reduced motor function over time are the hallmarks of normal aging. It has been hypothesized that modifiable factors (e.g., diet and exercise) may operate through three mechanisms: increasing cognitive reserve, decreasing the burden of vascular disease and decreasing stress [54]. Unhealthy aging as a result of poor diet, physical activity, or other behavioral and lifestyle factors, however, can give rise to debilitating neurodegenerative diseases, irrespective of any genetic predisposition. Inflammation and oxidative stress have been linked to premature cell death, lack of regeneration and impaired healing in different regions of the brain. A high-quality diet may increase endogenous anti-inflammatory protection in the brain and may decrease the loss of neuronal and behavioral function in senescence by providing adequate nutrients to counteract the effects of oxidative damage. 

Our study has several strengths. First, the HANDLS study recruited socioeconomically diverse African American adults who are often under-represented in large cohorts, particularly observational longitudinal studies. Second, diet quality was based on two 24 h recalls in contrast with other studies which rely on only one recall often with a far less comprehensive survey instrument. Third, our study used three different dietary indices to make a comprehensive measure of nutritional status of our study population. Fourth, this is the first study to look at reduced cognitive performance and increased genetic risk of AD in a predominantly African American cohort. Fifth, the genetic risk score was based on a comprehensive list of SNPs related to AD in the population, irrespective of race and sex. Fifth, despite the seemingly small sample size noted for various cognitive tests, we had enough power to test the associations; e.g., for verbal fluency test, we needed *N* = 84 African American participants to demonstrate 90% power, *N* = 63 for 80%. The study had *N* = 307 (total) and *N* = 162 (women) for observed results for this test that survived multiple testing. Lastly, the diet quality and cognitive function relationship has been examined in relation to individual SNPs in our study, which is also a first among African American urban adults. Therefore, the present study makes a unique contribution to the nutrition, genetic, and cognition literature, simultaneously.

Our study is not without limitations. Due to the cross-sectional nature of the analysis, we cannot infer causality. It is possible that reduced cognitive performance may precede poor diet quality. It is important to analyze the prospective association between changes in diet quality and cognitive performance, which is our next step. Another limitation is our reliance on participants’ recall assessing food consumption which is somewhat obviated by the consistency of recalls on two separate occasions. Although we performed our risk score calculation based on over one-hundred AD-related genes and reported SNPs, hundreds of more SNPs have been discovered since the Nature publication [32], and we are unable to claim our list as comprehensive. As shown in Supplementary material 3, the power to detect interaction effects might have been limited compared with that ascribed to main effects. It is possible that Type I errors might have influenced the observed associations. Finally, the study findings cannot be generalized to a population other than urban African Americans perhaps limited by restricting our recruitment to Baltimore City. 

What is presented here are preliminary findings, in a predominantly low SES population, that need further research. Future studies are necessary to test such associations in a larger, more heterogeneous samples with respect to diet quality, genetic risk, and cognition. It would also be interesting to identify factors that might contribute to low diet quality among higher-SES groups and examine how that correlates with a high genetic risk for AD. 

## 5. Conclusions

Overall, our findings give valuable insight into the effects of the modifiable (i.e., diet) vs. non-modifiable (genetics) factors on cognitive function in urban adults and how associations vary by sex. Although poor diet quality was not associated with poorer cognitive outcome among African American urban adults, it was influenced by their genetic risk for AD. Specifically, improved diet quality can modify performance on cognitive tests of verbal fluency among individuals with higher AD risk.

## Figures and Tables

**Table 1:** Individual single nucleotide polymorphisms (SNP) correlations with hAlzScore (genetic risk score for Alzheimer’s Disease (AD) in Healthy Aging in Neighborhoods of Diversity Across the Life Span (HANDLS)).

	hAlzScore	rs1049~C	rs1064~A	rs1659~T	rs1799~A	rs2251~T	rs2306~C	rs4055~A	rs4291_A	rs4343_A	rs4496~A	rs4806~C	rs4845~G
hAlzScore	1.00												
rs1049296_C	0.23	1.00											
rs1064039_A	0.32	0.03 *	1.00										
rs165932_T	0.32	0.03 *	0.03 *	1.00									
rs1799990_A	0.36	−0.007 *	0.02 *	0.06 *	1.00								
rs2251101_T	0.23	0.03 *	0.004 *	0.007 *	0.03 *	1.00							
rs2306604_C	0.40	0.02 *	0.05 *	0.01 *	0.01 *	0.05 *	1.00						
rs405509_A	0.16	−0.03 *	−0.03 *	−0.07 *	0.03 *	−0.07 *	0.06 *	1.00					
rs4291_A	0.37	0.02 *	−0.05 *	0.02 *	0.009 *	0.01 *	−0.01 *	−0.03 *	1.00				
rs4343_A	0.31	−0.001 *	−0.004 *	−0.04 *	−0.02 *	−0.02 *	−0.03 *	0.02 *	0.11	1.00			
rs449647_A	0.17	−0.01 *	0.04 *	−0.002 *	−0.03 *	0.01 *	−0.007 *	−0.43	0.03 *	−0.05 *	1.00		
rs4806173_C	0.33	−0.002 *	−0.004 *	0.02 *	−0.03 *	−0.03 *	0.03 *	−0.03 *	−0.004 *	0.02 *	0.0230 *	1.00	
rs4845378_G	0.21	0.03 *	−0.02 *	0.02 *	0.01 *	−0.006 *	0.01 *	0.02 *	0.02 *	0.01 *	−0.01 *	0.003 *	1.00

Genes/ SNPs: TF (rs1049296_C), CST3 (rs1064039_A), PSEN1 (rs165932_T), PRNP (rs1799990_A), IDE (rs2251101_T), TFAM (rs2306604_C), APOE-ε2 (rs405509_A), ACE (rs4291_A), ACE (rs4343_A), APOE- ε2 (rs449647_A), GAPDHS (rs4806173_C), and CHRNB2 (rs4845378_G).Note: SNP dosages that were reverse coded to create the hAlzScore, due to alternative allele increasing the risk of Alzheimer’s Disease.: rs165932, rs1799990, rs449647, rs4806173 and rs4845378; *, *p* < 0.05.

**Table 2:** Means and standard errors (Mean ± SE or %) by sex for selected characteristics of HANDLS participants ≥ 50 years old with complete Mini-Mental Status Examination (*n* = 304).

	All	Women	Men	*P* ^sex1^
	(*N* = 304)	(*N* = 163)	(*N* = 141)	
Age at baseline, years	56.90 ± 0.24	56.87 ± 0.33	56.93 ± 0.35	0.90
Education,	2.29 ± 0.03	2.29 ± 0.04	2.29 ± 0.05	0.96
Literacy (WRAT Score)	39.9 ± 0.46	40.37 ± 0.58	39.52 ± 0.73	0.36
Poverty Status<125%, %	44.4	46.6	41.8	0.40
Smoking Status, %	42.8 *	33.7 *	53.2 *	0.001 *
Use of illicit drugs, %	12.8 *	8.0 *	18.4 *	0.007 *
Body Mass Index, kg.m^−2^	30.46 ± 0.43 *	32.60 ± 0.65 *	27.99 ± 0.49 *	<0.001 *
hAlzScore	12.63 ± 0.10	12.67 ± 0.14	12.58 ± 0.16	0.70
HEI-total score	43.98 ± 0.65 *	45.64 ± 0.94 *	42.06 ± 0.86 *	0.006 *
**DASH-total score**	1.76 ± 0.08	2.01 ± 0.12	1.47 ± 0.09	0.40
**MAR-total score**	77.22 ± 1.29	76.20 ± 2.16	78.39 ± 1.25	0.40
Depressive Symptoms (CES-D Score)	14.67 ± 0.64	15.25 ± 0.94	14.00 ± 0.64	0.33
Diabetes; %	23.7	24.5	22.7	0.70
Hypertension; %	62.8 *	68.1 *	56.7 *	0.04 *
Dyslipidemia; %	36.5 *	41.1 *	31.2 *	0.07 *
Cardiovascular disease; %	24.7	26.4	22.7	0.45
Inflammatory conditions; %	19.4 *	26.4 *	11.4 *	0.001 *
NSAIDS; %	28.6	26.4	31.2	0.35
**Cognitive Test Scores**				
MMSE, (*N*)	27.04 ± 0.15, (304)	27.31 ± 0.18, (163)	26.73 ± 0.24, (141)	0.05 *
CVLT-List A, (*N*)	22.27 ± 0.34 *, (260)	23.40 ± 0.43 *, (147)	20.80 ± 0.51 *, (113)	<0.001 *
CVLT-DFR, (*N*)	22.50 ± 0.33 *, (253)	23.54 ± 0.42 *, (144)	21.12 ± 0.50 *, (109)	<0.001 *
BVRT, (*N*)	7.79 ± 0.33, (296)	7.94 ± 0.46, (159)	7.61 ± 0.47, (137)	0.61
Attention, (*N*)	5.96 ± 0.14, (271)	6.00 ± 0.19, (147)	5.92 ± 0.22, (124)	0.80
Trails A, (*N*)	49.08 ± 3.40, (300)	45.48 ± 3.75, (161)	53.25 ± 5.91, (139)	0.25
Trails B, (*N*)	226.14 ± 11.87, (300)	223.42 ± 16.03, (161)	229.28 ± 17.71, (139)	0.80
Digit Span Forward, (*N*)	6.74 ± 0.12, (294)	6.67 ± 0.16, (156)	6.81 ± 0.18, (138)	0.58
Digit Span Backward, (*N*)	5.08 ± 0.12, (292)	5.11 ± 0.16, (154)	5.04 ± 0.17, (138)	0.76
Clock Command, (*N*)	8.64 ± 0.07, (300)	8.64 ± 0.09, (162)	8.63 ± 0.10, (138)	0.94
Identical Pictures, (*N*)	20.38 ± 0.34, (230)	20.72 ± 0.46, (126)	19.96 ± 0.52, (104)	0.28
Card Rotation, (*N*)	29.03 ± 1.04 *, (233)	25.89 ± 1.36 *, (128)	32.84 ± 1.53 *, (105)	<0.001 *
Verbal fluency, (*N*)	17.34 ± 0.28 *, (301)	16.78 ± 0.34 *, (160)	17.97 ± 0.45 *, (141)	0.03 *

Abbreviations: hAlzScore = Alzheimer’s Risk Score; MMSE = Mini-Mental State Examination; CVLT-List A = California Verbal Learning test- List A; CVLT-DFR = California Verbal Learning Test-Delayed Free Recall; BVRT = Benton Visual Retention Test; Attention = Brief Test of Attention; Trails A = Trailmaking Test A; Trails B = Trailmaking Test B; Digit Span Forward = Digits Span Forward Test; Digit Span Backward = Digits Span Backward Test; Clock Command = Clock Command Test; Identical Pictures = Identical Pictures Test; Card Rotation = Card rotation Test; Verbal fluency = Verbal fluency Test. *P*^sex1^= *P*-value associated with null hypothesis of no difference by sex based on *t*-test for continuous variables and chi-square test for categorical variables.^2,^ Inverse mills ratio (mean ± SD) for the selected sample based on complete data on MMSE is 0.22 ± 5.31.3.2. Cognitive Tests and Their Association with hAlzScore and Individual SNPs of hAlzScore. *, *p* < 0.05.

**Table 3:** Associations between cognitive test performance and hAlzScore by sex, for HANDLS participants ≥50 year of age with complete and reliable cognitive test scores: Ordinary Least Square (OLS) regression models ^1^ (*p* < 0.05).

	All		Women		Men	
	β ± SE (*N*)	(*p*-Values)	β ± SE (*N*)	(*p*-Values)	β ± SE (*N*)	(*p*-Values)
*Mini-Mental State Exam, (MMSE)*						
hAlzScore	−0.01 ± 0.07 (304)	0.92	0.01 ± 0.09 (163)	0.93	0.03 ± 0.11 (141)	0.76
*California Verbal Learning Test (CVLT), List A*						
hAlzScore	−0.34 * ± 0.17 (267)	0.05	−0.42 * ± 0.24 (149)	0.08	−0.22 ± 0.27 (118)	0.43
*California Verbal Learning Test (CVLT), Free Recall Long Delay (FRLD)*						
hAlzScore	−0.44 ** ± 0.17 (261)	0.01	−0.48 ** ± 0.24 (146)	0.04	−0.39 ± 0.27 (115)	0.16
*Benton Visual Retention Test, (BVRT)*						
hAlzScore	0.37 ** ± 0.17 (302)	0.03	0.68 *** ± 0.25 (162)	0.007	0.04 ± 0.25 (140)	0.88
*Clock, Command*						
hAlzScore	−0.02 ± 0.04 (304)	0.61	−0.03 ± 0.05 (164)	0.53	0.01 ± 0.05 (140)	0.89
*Brief Test of Attention*						
hAlzScore	−0.06 ± 0.07 (277)	0.43	−0.16 ± 0.11 (149)	0.14	0.01 ± 0.11 (128)	0.36
*Trailmaking Test, Part A*						
hAlzScore	−2.861 ± 1.79 (312)	0.11	−3.45 ± 2.17 (164)	0.11	−4.96 ± 3.07 (148)	0.11
*Trailmaking Test, Part B*						
hAlzScore	−5.79 ± 5.50 (311)	0.29	0.01 * ± 8.38 (164)	0.10	−12.52 * ± 7.66 (147)	0.10
*Digits Span, Forward*						
hAlzScore	0.02 ± 0.06 (300)	0.77	−0.17 ± 0.09 (158)	0.84	0.10 ± 0.09 (142)	0.28
*Digits Span, Backward*						
hAlzScore	−0.04 *±* 0.05 (298)	0.51	−0.14 * *±* 0.08 (156)	0.06	0.08 *±* 0.08 (142)	0.32
*Card Rotation test*						
hAlzScore	0.08 *±* 0.56 (236)	0.89	0.40 *±* 0.81 (129)	0.62	−0.94 *±* 0.82 (107)	0.25
*Identical Pictures*						
hAlzScore	0.20 *±* 0.18 (233)	0.28	0.23 *±* 0.27 (127)	0.38	0.12 *±* 0.28 (106)	0.66
*Animal Fluency*						
hAlzScore	0.05 *±* 0.16 (307)	0.71	−0.12 *±* 0.19 (162)	0.53	0.26 *±* 0.24 (145)	0.28

Abbreviations: hAlzScore = Alzheimer’s Risk Score; MMSE = Mini-Mental State Examination; CVLT-List A = California Verbal Learning test- List A; CVLT-DFR = California Verbal Learning Test-Delayed Free Recall; BVRT = Benton Visual Retention Test; Attention = Brief Test of Attention; Trails A = Trailmaking Test A; Trails B = Trailmaking Test B; Digit Span Forward = Digits Span Forward Test; Digit Span Backward = Digits Span Backward Test; Clock Command = Clock Command Test; Identical Pictures = Identical Pictures Test; Card Rotation = Card rotation Test; Verbal fluency = Verbal fluency Test. ^1^ OLS regression models (for men and women combined and stratified by sex) were adjusted for age, sex, race, poverty status, education status, BMI, total energy intake, current smoking status, current drug use, depression, Diabetes, Hypertension, Dyslipidemia, Cardiovascular Disease, Inflammatory conditions and use of Non-Steroidal Anti-Inflammatory Drugs (NSAIDs).Continuous covariates were centered at their mean. ***, *p* < 0.01; **, *p* < 0.05, *, *p* < 0.10.

**Table 4:** Associations between cognitive test performance and selected dietary indices * (β ± SE, *p*-value), stratified by sex, for HANDLS participants ≥50 year of age: Ordinary Least Square OLS regression models ^1^ (*p* < 0.05).

	All	Women	Men
*Mini-Mental State Exam, MMSE*			
Model 1: HEI-2010	−0.01 ± 0.01, 0.60	0.002 ± 0.01, 0.86	−0.02 ± 0.02, 0.45
Model 2: DASH	0.02 ± 0.09, 0.80	−0.05 ± 0.10, 0.64	0.20 ± 0.18, 0.28
Model 3: MAR	0.002 ± 0.01, 0.77	0.004 ± 0.01, 0.60	−0.01 ± 0.02, 0.73
*California Verbal Learning Test CVLT, List A*			
Model 1: HEI-2010	−0.034 ± 0.03, 0.23	−0.01 ± 0.04, 0.89	−0.08 ± 0.05, 0.12
Model 2: DASH	−0.20 ± 0.24, 0.40	−0.14 ± 0.29, 0.63	−0.37 ± 0.44, 0.41
Model 3: MAR	−0.03 ± 0.02, 0.08	−0.02 ± 0.02, 0.34	−0.09 ± 0.04, 0.03
*California Verbal Learning Test (CVLT), Free Recall Long Delay (FRLD)*			
Model 1: HEI-2010	−0.03 ± 0.03, 0.28	0.00 ± 0.04, 0.99	−0.07 ± 0.05. 0.16
Model 2: DASH	−0.25 ± 0.24, 0.28	−0.14 ± 0.29, 0.63	−0.57 ± 0.44, 0.20
Model 3: MAR	−0.03 ± 0.02, 0.09	−0.02 ± 0.02, 0.44	−0.08 ± 0.04, 0.05
*Benton Visual Retention Test, BVRT*			
Model 1: HEI-2010	0.01 ± 0.03, 0.80	0.001 ± 0.04, 0.98	0.04 ± 0.05, 0.37
Model 2: DASH	−0.20 ± 0.25, 0.41	−0.28 ± 0.31, 0.36	0.16 ± 0.42, 0.71
Model 3: MAR	−0.04 ± 0.02, 0.03	−0.04 ± 0.02, 0.07	−0.02 ± 0.04, 0.68
*Clock, Command*			
Model 1: HEI-2010	−0.001 ± 0.01, 0.90	−0.01 ± 0.01, 0.26	0.01 ± 0.01, 0.23
Model 2: DASH	−0.05 ± 0.05, 0.31	−0.08 ± 0.07, 0.22	−0.01 ± 0.01, 0.91
Model 3: MAR	0.01 ± 0.004, 0.17	0.002 ± 0.004, 0.66	0.013 ± 0.01, 0.14
*Brief Test of Attention*			
Model 1: HEI-2010	−0.01 ± 0.01, 0.35	−0.01 ± 0.02, 0.39	−0.004 ± 0.02, 0.84
Model 2: DASH	−0.07 ± 0.10, 0.49	−0.08 ± 0.13, 0.53	−0.10 ± 0.18, 0.61
Model 3: MAR	0.02 ± 0.01, 0.03	0.01 ± 0.01, 0.11	0.03 ± 0.02, 0.09
*Trailmaking Test, Part A*			
Model 1: HEI-2010	0.01 ± 0.31, 0.97	0.01 ± 0.34, 0.98	−0.09 ± 0.60, 0.88
Model 2: DASH	−3.41 ± 2.55, 0.18	−1.71 ± 2.61, 0.51	−7.75 ± 5.37, 0.15
Model 3: MAR	−0.04 ± 0.18, 0.84	−0.01 ± 0.17, 0.96	0.07 ± 0.51, 0.90
*Trailmaking Test, Part B*			
Model 1: HEI-2010	0.02 ± 0.96, 0.98	−0.69 ± 1.31, 0.60	1.28 ± 1.49, 0.98
Model 2: DASH	7.95 ± 7.80, 0.31	15.34 ± 9.90, 0.12	−8.68 ± 13.48, 0.52
Model 3: MAR	0.09 ± 0.541, 0.87	−0.25 ± 0.65, 0.70	1.15 ± 1.26, 0.37
*Digits Span, Forward*			
Model 1: HEI-2010	0.02 ± 0.01, 0.06	0.02 ± 0.01, 0.17	0.02 ± 0.02, 0.29
Model 2: DASH	−0.07 ± 0.08, 0.40	0.02 ± 0.10, 0.85	−0.24 ± 0.16, 0.13
Model 3: MAR	0.01 ± 0.01, 0.33	0.01 ± 0.01, 0.29	0.002 ± 0.02, 0.88
*Digits Span, Backward*			
Model 1: HEI-2010	−0.01 ± 0.01, 0.21	−0.01 ± 0.01, 0.40	−0.01 ± 0.02, 0.60
Model 2: DASH	−0.07 ± 0.08, 0.34	−0.15 ± 0.10, 0.11	0.07 ± 0.14, 0.62
Model 3: MAR	0.003 ± 0.01, 0.54	0.01 ± 0.01, 0.31	0.001 ± 0.01, 0.97
*Card Rotation test*			
Model 1: HEI-2010	−0.02 ± 0.09, 0.81	−0.04 ± 0.12, 0.74	0.05 ± 0.16, 0.75
Model 2: DASH	0.26 ± 0.76, 0.74	−0.08 ± 0.94, 0.93	1.07 ± 1.32, 0.42
Model 3: MAR	0.05 ± 0.05, 0.31	0.03 ± 0.06, 0.65	0.21 ± 0.15, 0.16
*Identical Pictures*			
Model 1: HEI-2010	−0.01 ± 0.03, 0.69	-0.03 ± 0.04, 0.51	0.03 ± 0.06, 0.65
Model 2: DASH	0.07 ± 0.25, 0.77	0.08 ± 0.31, 0.81	0.04 ± 0.45, 0.93
Model 3: MAR	−0.01 ± 0.02, 0.56	-0.02 ± 0.02, 0.30	0.09 ± 0.05, 0.08
*Verbal fluency*			
Model 1: HEI-2010	−0.002 ± 0.03, 0.93	-0.01 ± 0.03, 0.72	0.001 ± 0.05, 0.99
Model 2: DASH	−0.11 ± 0.21, 0.61	-0.18 ± 0.23, 0.44	0.03 ± 0.42, 0.95
Model 3: MAR	0.01 ± 0.01, 0.41	0.01 ± 0.02, 0.55	0.02 ± 0.04, 0.58

Abbreviations: hAlzScore = Alzheimer’s Risk Score; MMSE = Mini-Mental State Examination; CVLT-List A = California Verbal Learning test- List A; CVLT-DFR = California Verbal Learning Test-Delayed Free Recall; BVRT = Benton Visual Retention Test; Attention = Brief Test of Attention; Trails A = Trailmaking Test A; Trails B = Trailmaking Test B; Digit Span Forward = Digits Span Forward Test; Digit Span Backward = Digits Span Backward Test; Clock Command = Clock Command Test; Identical Pictures = Identical Pictures Test; Card Rotation = Card rotation Test; Verbal fluency = Verbal fluency Test. ^1^ OLS regression models for men and women combined and stratified by sex were adjusted for age, sex, race, poverty status, education status, BMI, total energy intake, current smoking status, current drug use, depression, Diabetes, Hypertension, Dyslipidemia, Cardiovascular Disease, Inflammatory conditions and use of Non-Steroidal Anti-Inflammatory Drugs NSAIDs. Models 1–3 had main exposure variables HEI-2010, DASH and MAR, respectively. Continuous covariates were centered at their mean. * Sample sizes overall and by sex for each cognitive test outcome can be found in Table 2.

**Table 5:** Associations of cognitive performance test scores ^1^ with 2-way interactions of hAlzScore and selected dietary indices, stratified by sex, for HANDLS participants ≥50y of age (β ± SE, *p*-value): Ordinary Least Square, OLS regression models.^2^ (*p* < 0.10).

	All	Women	Men
***Clock, Command***			
Model 1: hAlzScore × HEI2010	0.01 ± 0.003, 0.10	0.01 ± 0.004, 0.17	0.003 ± 0.01, 0.46
Model 2: hAlzScore × DASH	0.02 ± 0.03, 0.49	0.02 ± 0.03, 0.57	0.01 ± 0.05, 0.88
Model 3: hAlzScore × MAR	0.003 ± 0.001, 0.13	0.01 ± 0.002, 0.04 **	−0.003 ± 0.0003, 0.38
***Card Rotation test***			
Model 1: hAlzScore × HEI2010	0.11 ± 0.05, 0.04 **	0.10 ± 0.07, 0.13	0.08 ± 0.10, 0.44
Model 2: hAlzScore × DASH	0.45 ± 0.44, 0.31	0.73 ± 0.54, 0.18	−0.59 ± 0.83, 0.47
Model 3: hAlzScore × MAR	−0.02 ± 0.03, 0.56	−0.02 ± 0.04, 0.63	0.03 ± 0.07, 0.66
***Identical Pictures***			
Model 1: hAlzScore × HEI2010	0.03 ± 0.02, 0.07	0.02 ± 0.02, 0.31	0.05 ± 0.03, 0.13
Model 2: hAlzScore × DASH	0.14 ± 0.15, 0.35	0.25 ± 0.18, 0.16	−0.10 ± 0.28, 0.71
Model 3: hAlzScore × MAR	0.004 ± 0.01, 0.74	0.002 ± 0.01, 0.85	0.02 ± 0.02, 0.39
***Verbal fluency***			
Model 1: hAlzScore × HEI2010	0.03 ± 0.01, 0.02 **	0.04 ± 0.02, 0.02 **	0.02 ± 0.02, 0.37
Model 2: hAlzScore × DASH	0.18 ± 0.11, 0.09	0.25 ± 0.12, 0.04 **	−0.01 ± 0.22, 0.98
Model 3: hAlzScore × MAR	0.01 ± 0.01, 0.32	0.01 ± 0.01, 0.51	0.01 ± 0.02, 0.71

Abbreviations: hAlzScore = Alzheimer’s Risk Score; MMSE = Mini-Mental State Examination; CVLT-List A = California Verbal Learning test- List A; CVLT-FRLD = California Verbal Learning Test- Free Recall Long Delayed (FRLD); BVRT = Benton Visual Retention Test; Attention = Brief Test of Attention; Trails A = Trailmaking Test A; Trails B = Trailmaking Test B; Digit Span Forward = Digits Span Forward Test; Digit Span Backward = Digits Span Backward Test; Clock Command = Clock Command Test; Identical Pictures = Identical Pictures Test; Card Rotation = Card rotation Test; Verbal fluency = Verbal fluency Test. (** *p* < 0.05). ^1^ Tests that did not have significant hAlzScore interactions are: MMSE, CVLT List-A, CVLT FRLD, BVRT, Trails A, Trails B, Digits Span Forwards, and Digits Span Backwards, and therefore omitted from the table. Complete hAlzScore interaction analyses can be found in Supplementary Table S3(d). ^2^ OLS regression models, for men and women combined and stratified by sex were adjusted for age, sex, race, poverty status, education status, BMI, total energy intake, current smoking status, current drug use, depression, Diabetes, Hypertension, Dyslipidemia, Cardiovascular Disease, Inflammatory conditions and use of Non-Steroidal Anti-Inflammatory Drugs, NSAIDs. Covariates were centered at the mean. 2-way interaction terms were added for hAlzScore and dietary quality indices. Main effects of those exposures were included along with main effects of covariates. Sample sizes for each model and stratum can be found in Table 2.

## Supplementary Materials

The following are available online at https://www.mdpi.com/2072-6643/11/9/2181/s1, Table S1: Seventy SNPs from AlzGene Database genotyped and/or imputed in the HANDLS African American subjects; Table S2: Cognitive performance test scores (by sex), for HANDLS participants ≥ 50 year by individual SNPs; Table S3(a). Associations of cognitive performance test scores with 2-way interactions of AlzScore and selected dietary indices, stratified by sex, for HANDLS participants ≥50 year of age (β ± SE, *p*-value): Ordinary Least Square, OLS regression models.^1^ Table S3 (b): Two-way interaction (*p*-values) of cognitive performance test scores, select SNP^1§^s and DASH^2^ by sex at baseline, for HANDLS participants ≥ 50 year of age; Table S3 (c): Two-way interaction (*p*-values) of cognitive performance test scores, select SNP^1§^s and MAR^2^ by sex at baseline, for HANDLS participants ≥ 50 year of age; Table S3 (d): Two-way interaction (*p*-values) of cognitive performance test scores, select SNP^1§^s and HEI-2010 by sex at baseline, for HANDLS participants ≥ 50 year of age; Supplementary material 1: Cognitive Tests; Supplementary material 2: Diet Quality Interaction Supplementary material 3: Power Calculation.
