# Supplementary material for: The Interplay of Diet Quality and Alzheimer’s Disease Genetic Risk Score in Relation to Cognitive Performance Among Urban African Americans
Source: Nutrients. 2019 Sep 11;11(9):2181. doi: 10.3390/nu11092181 (PMC6769979; doi:10.3390/nu11092181)
Supplement: Supplementary file 1 [file nutrients-11-02181-s001.pdf]

Table S1: Seventy SNPs from AlzGene Database genotyped and/or imputed in the HANDLS African American subjects

**Single Nucleotide Polymorphisms (SNPs)**	**Genes**	Al1 (Major)	Al2 (Minor)	Frequency
TF*	rs1049296	C	T	0.91758
BLMH	rs1050565	T	C	0.79546
CST3*	rs1064039	C	T	0.7903
IL1B	rs1143634	G	A	0.87227
LDLR	rs11669576	G	A	0.81711
OLR1	rs12316150	A	T	0.9716
APBB2	rs13133980	G	C	0.76012
MAPT	rs1467967	A	G	0.63136
PSEN1*	rs165932	T	G	0.74596
CTSD	rs17571	G	A	0.96512
TNF	rs1799724	C	T	0.9671
CCR2	rs1799864	G	A	0.84532
DLST	rs1799900	A	G	0.9126
HFE	rs1799945	C	G	0.97464
NOS3	rs1799983	G	T	0.90184
LRP1	rs1799986	C	T	0.93883
PRNP*	rs1799990	A	G	0.66874
TGFB1	rs1800469	G	A	0.75953

HFE	rs1800562	G	A	0.99267
IL1A	rs1800587	G	A	0.60793
TNF	rs1800629	G	A	0.87327
FAS	rs1800682	G	A	0.70754
IL6	rs1800795	G	C	0.92192
IL10	rs1800871	G	A	0.59249
IL10	rs1800872	G	T	0.5916
IL10	rs1800896	T	C	0.65699
MTHFR	rs1801131	T	G	0.82483
MTHFR	rs1801133	G	A	0.91162
TCN2	rs1801198	C	G	0.77133
BCHE	rs1803274	C	T	0.82747
HTR6	rs1805054	C	T	0.8273
IDE	rs1832196	G	A	0.73245
CHAT	rs1880676	G	A	0.95166
IDE*	rs1887922	T	C	0.91934
TFAM*	rs1937	G	C	0.97402
IDE	rs1999764	T	C	0.90194
PLAU	rs2227564	C	T	0.9595
ABCA1	rs2230806	C	T	0.36582
ABCA1	rs2230808	C	T	0.234
IDE	rs2251101	T	C	0.88928

TFAM	rs2306604	G	A	0.80018
MPO	rs2333227	C	T	0.67856
SLC6A4	rs25531	T	C	0.78126
LPL	rs268	A	G	0.9978
LPL	rs328	C	G	0.93071
NOTCH4	rs367398	G	A	0.50259
IDE	rs3758505	A	C	0.70459
CHAT	rs3810950	G	A	0.95166
APOE*	rs405509	G	T	0.72841
PLAU	rs4065	C	T	0.52426
ACE*	rs4291	A	T	0.67994
ACE*	rs4343	A	G	0.76874
APOE*	rs449647	A	T	0.71431
IDE	rs4646953	A	G	0.90724
IDE	rs4646954	G	A	0.70117
GAPDHS*	rs4806173	C	G	0.68382
CHRNB2*	rs4845378	G	T	0.93035
CYP46A1	rs4900442	C	T	0.67679
ICAM1	rs5498	A	G	0.80201
IDE	rs551266	T	C	0.91951
BDNF	rs56164415	G	A	0.94897
BDNF	rs6265	C	T	0.96873

HTR2A	rs6313	G	A	0.60939
BACE1	rs638405	G	C	0.5253
PON1	rs662	C	T	0.64648
TNF	rs673	G	A	0.94328
CETP	rs708272	G	A	0.73507
CYP46A1	rs754203	A	G	0.85181
APOE	rs769446	T	C	0.94834
PSEN2	rs8383	C	T	0.58134

*SNPs used to create AlzScore in HANDLS

SNP details: rs1049296_C "TF (C>T)"; rs1064039_A "CST3 (A>G)" ; rs165932_Tinv "PSEN1 (G>T)"; rs1799990_Ainv "PRNP (G>A)"; rs2251101_T "IDE (T>C)"; rs2306604_C "TFAM (C>T)"; rs405509_A "APOE (A>C)"; rs4291_A "ACE (A>T)"; rs4343_A "ACE (A>G)"; rs449647_Ainv "APOE (T>A)"; rs4806173_Cinv "GAPDHS (G>C)"; rs4845378_Ginv "CHRNB2(T>G)"

Table S2. Demographic characteristics of the excluded and included participants for analyses.

	**Excluded***	**Anlyzed**	$\mathbf{P^{diff}}$
	(*N* = 1,349)	(*N* = 316)	
Age at baseline, y	56.87 ± 0.12	56.93 ± 0.24	0.84
Education, %			
<HS	9.49	7.28	**0.001**
HS	56.12	55.06	
>HS	30.84	37.66	
Poverty Status<125% , %	37.29	44.62	**0.02**
Sex, %women	55.60	52.53	0.32

Note: *The target population was African American participants above the age of 50 years (N=1,665). Out of those only 316 had complete data on genetics, dietary and cognition. MMSE had the most complete data among all cognitive tests and was used for baseline demographic comparison. P^{diff}= Based on t-test of null hypothesis of no difference between groups (analyzed vs. excluded). HS= High School.

Table S3. Cognitive performance test scores (by sex), for HANDLS participants ≥ 50y by individual SNPs.

	**All**	**Women**	**Men**
Mini-Mental State Exam, total score			
rs1049296_C	0.022±0.330 (0.95)	-0.290±0.401 (0.47)	0.621±0.579 (0.29)
rs1064039_A	0.199± 0.203 (0.33)	-0.044± 0.278 (0.87)	0.512± 0.311(0.10)
rs165932_T	-0.055± 0.213 (0.80)	0.179± 0.288 (0.54)	-0.168± 0.327 (0.61)
rs1799990_A	-0.304± 0.195 (0.12)	-0.243± 0.250 (0.33)	-0.440± 0.322 (0.17)
rs2251101_T	-0.194± 0.294 (0.51)	-0.280± 0.434 (0.52)	-0.147± 0.429 (0.73)
rs2306604_C	-0.285± 0.222 (0.20)	-0.276± 0.301 (0.36)	-0.310± 0.319 (0.38)
rs405509_A	**-0.334± 0.196 (0.09*)**	-0.305± 0.244 (0.21)	0.004± 0.336 (0.99)
rs4291_A	**-0.403± 0.200 (0.045**)**	0.041± 0.267 (0.88)	**-0.733± 0.303 (0.017**)**
rs4343_A	-0.018± 0.209 (0.93)	-0.131± 0.265 (0.62)	0.070± 0.347 (0.84)
rs449647_A	**-0.522± 0.203 (0.01**)**	**-1.021± 0.254 (0.000***)**	0.017± 0.336 (0.96)
rs4806173_C	0.042± 0.196 (0.83)	0.243± 0.246 (0.33)	-0.216± 0.336 (0.52)
rs4845378_G	-0.023± 0.395 (0.95)	-0.508± 0.464 (0.28)	0.692± 0.703 (0.32)
California Verbal Learning Test (CVLT), List A			
rs1049296_C	0.763± 0.872 (0.38)	-0.421±1.083 (0.70)	2.317± 1.673 (0.17)
rs1064039_A	0.079± 0.512 (0.88)	-0.159± 0.732 (0.83)	0.533± 0.795 (0.50)

rs165932_T	0.059± 0.523 (0.90)	-0.136±0.107 (0.86)	0.384± 0.798 (0.63)
rs1799990_A	-0.264± 0.480 (0.58)	-0.478± 0.676 (0.48)	0.080± 0.777 (0.92)
rs2251101_T	-0.943± 0.741 (0.20)	-1.340± 1.150 (0.25)	-0.679± 1.075 (0.53)
rs2306604_C	-0.058± 0.576 (0.92)	-0.778± 0.814 (0.34)	1.179± 0.903 (0.20)
rs405509_A	-0.567± 0.495 (0.25)	-0.909± 0.648 (0.16)	0.003± 0.857 (0.99)
rs4291_A	-0.542± 0.502 (0.28)	-0.649± 0.711 (0.36)	-0.442± 0.757 (0.56)
rs4343_A	-0.712± 0.515 (0.17)	-0.400± 0.697 (0.57)	-1.321± 0.836 (0.12)
rs449647_A	0.695± 0.507 (0.17)	0.290± 0.715 (0.69)	0.969± 0.798 (0.23)
rs4806173_C	0.580± 0.481 (0.23)	0.450± 0.656 (0.49)	0.843± 0.859 (0.33)
rs4845378_G	-0.645± 1.007 (0.52)	-0.199± 1.340 (0.88)	-1.682± 1.690 (0.32)
California Verbal Learning Test (CVLT), Delayed Free Recall			
rs1049296_C	0.669± 0.875 (0.45)	-0.289± 1.071 (0.79)	1.994± 1.732 (0.25)
rs1064039_A	-0.111± 0.510 (0.83)	-0.340± 0.731 (0.64)	0.199± 0.786 (0.80)
rs165932_T	0.190± 0.520 (0.72)	0.012± 0.754 (0.99)	0.402± 0.782 (0.61)
rs1799990_A	-0.411±0.479 (0.39)	-0.570± 0.671 (0.40)	-0.062± 0.783 (0.94)
rs2251101_T	-1.105± 0.739 (0.14)	-1.145± 1.140 (0.32)	-1.295± 1.069 (0.23)
rs2306604_C	-0.094± 0.576 (0.87)	-0.720± 0.813 (0.38)	1.151± 0.890 (0.20)
rs405509_A	-0.522± 0.494 (0.29)	-0.867± 0.647 (0.18)	-0.077± 0.852 (0.93)
rs4291_A	-0.768± 0.500 (0.13)	-0.849± 0.708 (0.23)	-0.807± 0.753 (0.29)
rs4343_A	-0.825± 0.514 (0.11)	-0.594± 0.695 (0.39)	**-1.432± 0.827 (0.09*)**
rs449647_A	**0.878± 0.502 (0.08*)**	0.391± 0.709 (0.58)	1.222± 0.786 (0.12)

rs4806173_C	0.587± 0.482 (0.23)	0.502± 0.649 (0.44)	0.573± 0.854 (0.50)
rs4845378_G	-0.442± 1.005 (0.66)	-0.275± 1.349 (0.84)	-1.033± 1.669 (0.54)
Benton Visual Retention Test, (BVRT)			
rs1049296_C	0.577± 0.890 (0.52)	1.288± 1.199 (0.29)	0.146± 1.377 (0.92)
rs1064039_A	-0.121± 0.525 (0.82)	0.720± 0.794 (0.37)	-0.577± 0.708 (0.42)
rs165932_T	0.870± 0.557 (0.12)	0.694± 0.847 (0.41)	0.924± 0.739 (0.21)
rs1799990_A	-0.608± 0.525 (0.25)	-0.974± 0.756 (0.62)	-0.684± 0.750 (0.36)
rs2251101_T	1.005± 0.771 (0.19)	0.331± 1.284 (0.80)	0.971± 0.972 (0.32)
rs2306604_C	**1.089± 0.582 (0.06*)**	**2.205± 0.870 (0.01**)**	-0.091± 0.803 (0.91)
rs405509_A	-0.329± 0.515 (0.52)	-0.324± 0.718 (0.65)	-0.448± 0.759 (0.56)
rs4291_A	**1.490± 0.527 (0.005***)**	**1.550± 0.780 (0.05**)**	**1.237± 0.707 (0.08*)**
rs4343_A	0.333± 0.551 (0.55)	0.418± 0.781 (0.59)	0.040± 0.789 (0.96)
rs449647_A	**-0.897± 0.536 (0.10*)**	-0.822± 0.768 (0.29)	-0.919± 0.763 (0.23)
rs4806173_C	-0.313± 0.522 (0.55)	**-1.211± 0.725 (0.10*)**	0.325± 0.789 (0.68)
rs4845378_G	**1.878± 1.033 (0.07*)**	0.828± 1.376 (0.55)	**3.837± 1.564 (0.02**)**
Clock, Command			
rs1049296_C	0.204± 0.182 (0.265)	-0.061±0.253 (0.81)	**0.657± 0.292 (0.03**)**
rs1064039_A	0.040± 0.111 (0.72)	0.065± 0.175 (0.70)	-0.001± 0.155 (0.99)
rs165932_T	-0.022± 0.117 (0.85)	0.267± 0.181 (0.14)	**-0.277± 0.162 (0.09*)**
rs1799990_A	0.072± 0.108 (0.51)	-0.060± 0.157 (0.70)	0.249± 0.163 (0.13)
rs2251101_T	-0.227± 0.160 (0.16)	-0.142± 0.272 (0.60)	-0.274± 0.212 (0.20)
rs2306604_C	0.116± 0.122 (0.34)	0.271± 0.189 (0.15)	0.021± 0.176 (0.91)

rs405509_A	0.148± 0.107 (0.17)	0.058± 0.154 (0.70)	**0.296± 0.165 (0.08*)**
rs4291_A	-0.073± 0.111 (0.51)	-0.070± 0.168 (0.68)	-0.061± 0.157 (0.70)
rs4343_A	-0.028± 0.115 (0.80)	-0.100± 0.166 (0.55)	0.036± 0.175 (0.84)
rs449647_A	0.023± 0.113 (0.84)	-0.101± 0.167 (0.55)	0.121± 0.168 (0.47)
rs4806173_C	**0.237± 0.108 (0.03**)**	**0.255± 0.153 (0.10*)**	0.265± 0.174 (0.13)
rs4845378_G	0.019± 0.217 (0.93)	0.172± 0.293 (0.56)	-0.376± 0.351 (0.29)
Brief Test of Attention			
rs1049296_C	-0.186± 0.374 (0.62)	-0.589± 0.495 (0.24)	0.244± 0.638 (0.70)
rs1064039_A	0.004± 0.217 (0.98)	-0.053± 0.327 (0.87)	0.229± 0.311 (0.46)
rs165932_T	**-0.001± 0.232 (0.10*)**	0.422± 0.356 (0.24)	-0.438± 0.310 (0.16)
rs1799990_A	0.274± 0.210 (0.19)	0.241± 0.303 (0.43)	0.449± 0.307 (0.15)
rs2251101_T	0.081± 0.315 (0.80)	-0.153± 0.529 (0.77)	0.008± 0.408 (0.98)
rs2306604_C	-0.066± 0.243 (0.79)	0.283± 0.375 (0.45)	-0.254± 0.341 (0.46)
rs405509_A	0.206± 0.217 (0.34)	**0.605± 0.304 (0.05**)**	-0.255± 0.329 (0.44)
rs4291_A	-0.052± 0.222 (0.81)	-0.052± 0.333 (0.88)	-0.018± 0.304 (0.95)
rs4343_A	0.188± 0.231 (0.42)	-0.057± 0.322 (0.86)	0.515± 0.348 (0.14)
rs449647_A	0.345± 0.220 (0.12)	**0.832± 0.321 (0.01**)**	-0.256± 0.320 (0.43)
rs4806173_C	0.210± 0.211 (0.32)	0.265± 0.298 (0.38)	-0.023± 0.339 (0.95)
rs4845378_G	-0.379± 0.430 (0.38)	0.338± 0.588 (0.57)	**-1.476± 0.650 (0.03**)**
Trailmaking Test, Part A			
rs1049296_C	-8.733± 8.973 (0.33)	2.89± 10.064 (0.77)	**-31.148± 16.770 (0.07**)**
rs1064039_A	-5.677± 5.422 (0.30)	-6.600± 6.741 (0.33)	-9.269± 9.049 (0.31)

rs165932_T	2.024± 5.793 (0.73)	-0.844± 7.281 (0.91)	4.060± 9.517 (0.67)
rs1799990_A	4.980± 5.292 (0.35)	**10.380±6.268 (0.10*)**	1.048± 9.267 (0.91)
rs2251101_T	-2.432± 7.959 (0.76)	2.503± 10.911 (0.82)	-6.393± 12.418 (0.61)
rs2306604_C	-2.108± 6.083 (0.73)	4.318± 7.593 (0.57)	-9.234± 10.268 (0.37)
rs405509_A	4.768± 5.329 (0.37)	4.943± 6.127 (0.42)	**-0.141± 9.741 (0.10*)**
rs4291_A	-3.944± 5.490 (0.47)	**-14.656± 6.609 (0.03**)**	4.069± 9.061 (0.65)
rs4343_A	-6.523± 5.642 (0.25)	-8.955± 6.636 (0.18)	-5.038± 9.907 (0.61)
rs449647_A	6.895± 5.529 (0.21)	6.868± 6.556 (0.30)	10.640± 9.647 (0.27)
rs4806173_C	-5.551± 5.310 (0.30)	-3.967± 6.223 (0.53)	-5.724± 9.750 (0.56)
rs4845378_G	6.138± 10.817 (0.57)	0.875± 11.721 (0.94)	23.618± 20.761 (0.26)
Trailmaking Test, Part B			
rs1049296_C	-29.379± 27.421 (0.29)	-15.039± 38.490 (0.70)	-36.896± 42.170 (0.38)
rs1064039_A	**-29.603± 16.540 (0.08*)**	10.627± 25.857 (0.68)	**-63.235± 22.014 (0.005***)**
rs165932_T	-10.257± 17.752 (0.55)	-24.252± 27.781 (0.38)	9.250± 23.815 (0.70)
rs1799990_A	-9.950± 16.278 (0.54)	-30.755± 24.070 (0.20)	5.698± 23.495 (0.81)
rs2251101_T	-17.011± 24.345 (0.49)	**-0.109± 41.747 (0.10*)**	-27.414± 30.972 (0.38)
rs2306604_C	**-30.468± 18.570 (0.10*)**	**-62.514± 28.610 (0.03**)**	8.897± 25.778 (0.73)
rs405509_A	-8.763± 16.319 (0.59)	-31.186± 23.350 (0.18)	23.292± 24.198 (0.34)
rs4291_A	-10.263± 16.826 (0.54)	2.062± 25.705 (0.94)	-18.001± 22.663 (0.43)
rs4343_A	8.477±17.294 (0.62)	-1.778± 25.542 (0.95)	10.398± 24.732 (0.68)
rs449647_A	-20.289± 16.911 (0.23)	-23.772± 25.097 (0.35)	-7.799± 24.134 (0.75)
rs4806173_C	-6.725± 16.260 (0.68)	-21.785± 23.769 (0.36)	24.053± 24.209 (0.32)

rs4845378_G	**59.716± 32.912 (0.07*)**	67.983± 44.480 (0.13)	50.811± 52.006 (0.33)
Digits Span, Forward			
rs1049296_C	**-0.564± 0.290 (0.05**)**	**-0.648± 0.367 (0.08*)**	-0.754± 0.497 (0.13)
rs1064039_A	0.139± 0.177 (0.43)	0.067± 0.252 (0.79)	0.227± 0.265 (0.39)
rs165932_T	**-0.594± 0.182 (0.001***)**	**-0.589± 0.264 (0.03**)**	**-0.655± 0.268 (0.02**)**
rs1799990_A	0.105± 0.169 (0.53)	-0.044± 0.236 (0.85)	0.363± 0.265 (0.17)
rs2251101_T	0.074± 0.265 (0.77)	-0.086± 0.403 (0.83)	0.342± 0.359 (0.34)
rs2306604_C	-0.162± 0.194 (0.40)	0.177± 0.281 (0.53)	-0.341± 0.293 (0.25)
rs405509_A	-0.017± 0.172 (0.92)	0.049± 0.228 (0.83)	-0.091± 0.283 (0.75)
rs4291_A	**-0.377± 0.174 (0.03**)**	**-0.887± 0.240 (0.000***)**	0.085± 0.262 (0.75)
rs4343_A	0.070± 0.183 (0.70)	-0.010± 0.247 (0.97)	0.307± 0.288 (0.29)
rs449647_A	0.132± 0.180 (0.46)	0.248± 0.247 (0.32)	-0.076± 0.279 (0.79)
rs4806173_C	**-0.378± 0.170 (0.03**)**	**-0.434± 0.227 (0.06*)**	-0.384± 0.285 (0.18)
rs4845378_G	0.140± 0.344 (0.68)	0.086± 0.431 (0.84)	-0.014± 0.596 (0.98)
Digits Span, Backward			
rs1049296_C	0.152± 0.277 (0.58)	-0.182± 0.358 (0.61)	0.472± 0.452 (0.30)
rs1064039_A	0.016± 0.166 (0.92)	-0.141± 0.239 (0.56)	0.173± 0.240 (0.47)
rs165932_T	**-0.336± 0.174 (0.06**)**	-0.082± 0.256 (0.75)	**-0.595± 0.242 (0.02**)**
rs1799990_A	0.216± 0.159 (0.18)	0.173± 0.224 (0.44)	0.271± 0.241 (0.26)
rs2251101_T	0.068± 0.240 (0.79)	-0.385± 0.385 (0.32)	0.354± 0.325 (0.28)
rs2306604_C	**-0.484± 0.180 (0.008***)**	-0.286± 0.267 (0.29)	**-0.648± 0.260 (0.01**)**
rs405509_A	-0.005± 0.162 (0.98)	0.224± 0.216 (0.30)	-0.248± 0.255 (0.33)

rs4291_A	-0.013± 0.165 (0.94)	-0.221± 0.238 (0.35)	0.113± 0.237 (0.63)
rs4343_A	-0.317± 0.174 (0.86)	-0.276± 0.240 (0.25)	0.256± 0.261 (0.33)
rs449647_A	0.162± 0.169 (0.34)	0.318± 0.234 (0.18)	-0.043± 0.253 (0.87)
rs4806173_C	0.069± 0.161 (0.67)	0.286± 0.217 (0.19)	-0.221± 0.259 (0.40)
rs4845378_G	**-0.670± 0.324 (0.04**)**	**-0.696± 0.411 (0.09*)**	-0.453± 0.538 (0.40)
Card Rotation test			
rs1049296_C	2.538± 2.786 (0.36)	1.391± 3.669 (0.71)	4.990± 4.945 (0.32)
rs1064039_A	1.289± 1.570 (0.41)	2.379± 2.326 (0.31)	-0.866± 2.326 (0.71)
rs165932_T	-0.951± 1.652 (0.57)	-0.943± 2.413 (0.70)	-1.386± 2.328 (0.55)
rs1799990_A	0.883± 1.557 (0.57)	0.064± 2.159 (0.98)	**4.486± 2.434 (0.07*)**
rs2251101_T	1.304± 2.404 (0.59)	3.187± 3.759 (0.40)	-1.838± 3.242 (0.57)
rs2306604_C	-1.505± 1.852 (0.42)	-0.424± 2.940 (0.89)	-4.161± 2.576 (0.11)
rs405509_A	-1.030± 1.609 (0.52)	-1.805± 2.270 (0.43)	-0.924± 2.426 (0.70)
rs4291_A	-1.192± 1.667 (0.48)	-1.191± 2.3098 (0.62)	-1.297± 2.425 (0.59)
rs4343_A	0.972± 1.665 (0.56)	0.898± 2.304 (0.70)	0.542± 2.534 (0.83)
rs449647_A	-0.032± 1.641 (0.98)	-1.853± 2.377 (0.44)	2.999± 2.392 (0.21)
rs4806173_C	0.380± 1.606 (0.81)	1.809± 2.201 (0.41)	-3.341± 2.626 (0.21)
rs4845378_G	-2.454± 3.155 (0.44)	-3.322± 4.170 (0.43)	-4.463± 5.147 (0.39)
Identical Pictures			
rs1049296_C	0.749± 0.922 (0.42)	**2.432± 1.193 (0.04**)**	-0.762± 1.684 (0.65)
rs1064039_A	0.538± 0.526 (0.31)	0.287± 0.778 (0.71)	0.789± 0.796 (0.32)
rs165932_T	-0.647± 0.547 (0.24)	-0.104± 0.804 (0.90)	**-1.464± 0.775 (0.06*)**

rs1799990_A	-0.237± 0.516 (0.65)	**-1.533± 0.701 (0.03**)**	**1.586± 0.823 (0.06*)**
rs2251101_T	0.233± 0.806 (0.77)	0.404± 1.261 (0.75)	-0.526± 1.120 (0.64)
rs2306604_C	-0.134± 0.616 (0.83)	-0.461± 0.980 (0.64)	-0.684± 0.883 (0.44)
rs405509_A	-0.132± 0.534 (0.81)	0.770± 0.754 (0.31)	-1.267± 0.812 (0.12)
rs4291_A	0.285± 0.553 (0.61)	-0.265± 0.797 (0.74)	0.783± 0.819 (0.34)
rs4343_A	-0.189± 0.553 (0.73)	-0.909± 0.764 (0.34)	0.479± 0.859 (0.58)
rs449647_A	0.104± 0.545 (0.85)	0.306± 0.794 (0.70)	0.047± 0.817 (0.95)
rs4806173_C	0.133± 0.536 (0.80)	0.470± 0.735 (0.52)	-1.017± 0.907 (0.27)
rs4845378_G	-1.028± 1.044 (0.33)	-0.317± 1.388 (0.82)	-2.262± 1.736 (0.20)
Animal Fluency			
rs1049296_C	0.611± 0.730 (0.40)	-0.373± 0.901 (0.68)	**2.153± 1.298 (0.10*)**
rs1064039_A	0.386± 0.440 (0.38)	0.243± 0.600 (0.69)	0.375± 0.703 (0.59)
rs165932_T	0.040± 0.468 (0.93)	0.793± 0.641 (0.22)	-0.588± 0.725 (0.42)
rs1799990_A	0.155± 0.430 (0.72)	0.281± 0.556 (0.61)	-0.171± 0.717 (0.81)
rs2251101_T	**-1.070± 0.639 (0.10*)**	-0.914± 0.956 (0.34)	-1.177± 0.951 (0.22)
rs2306604_C	**0.867± 0.487 (0.08*)**	0.544± 0.673 (0.42)	0.815± 0.780 (0.30)
rs405509_A	-0.689± 0.431 (0.11)	-0.460± 0.544 (0.40)	-0.943± 0.745 (0.21)
rs4291_A	-0.198± 0.443 (0.66)	-0.592± 0.591 (0.32)	0.223± 0.693 (0.75)
rs4343_A	0.064± 0.460 (0.90)	0.262± 0.589 (0.66)	**-0.118± 0.773 (0.10*)**
rs449647_A	-0.645± 0.444 (0.15)	**-0.402± 0.590 (0.50**)**	-1.013± 0.733 (0.17)
rs4806173_C	-0.423± 0.430 (0.33)	-0.177± 0.547 (0.75)	-0.996± 0.752 (0.19)
rs4845378_G	1.339± 0.872 (0.13)	0.112± 1.046 (0.91)	**3.344± 1.554 (0.03**)**

Notes: 1. Results are shown as β-coefficients ± Standard Errors, (p-value) *** p<0.01, ** p<0.05, * p<0.1

2. SNPs inverted to match the directionality of increased risk are: rs165932, rs1799990, rs449647, rs4806173 and rs4845378

3. The total number of cognitive test associations by individual SNPs of corresponding genes in at least one group (all, women, men) are as follows: TF, rs1049296 (n = 5); CST3, rs1064039 (n = 1); PSEN, rs165932 (n = 5); PRNP, rs1799990 (n = 3); IDE, rs2251101 (n = 2); TFAM, rs2306604 (n = 4); APOE, rs405509 (n = 4); ACE, rs4291 (n = 4); ACE, rs4343 (n = 1); APOE, rs449647 (n = 5); GAPDHS, rs4806173(n = 2) and CHRNB2, rs4845378 (n = 5). However, only two of those associations survived correction for multiple testing. Among women, MMSE was inversely related to rs449647 ("A" allele), while Digits Span Forward, was inversely related to rs165932 ("T" allele) in the total population and to rs4291 ("A" allele) among women ($p < 0.004$).

Table S4. (a). Associations of cognitive performance test scores with 2-way interactions of AlzScore and selected dietary indices, stratified by sex, for HANDLS participants ≥50y of age [β±SE, p-value]: Ordinary Least Square, OLS regression models.[1.]

	All	Women	Men
Mini-Mental State Exam, , MMSE			
Model 1: AlzScore	-0.01±0.1, 0.91	0.01±0.1, 0.93	0.10±0.1, 0.63
Model 1: HEI2010	-0.01±0.01, 0.51	0.0±0.01, 0.86	-0.02±0.02, 0.30
Model 1: AlzScore×HEI2010	0.01±0.01, 0.29	0.0±0.01, 0.10	0.01±0.01, 0.18
Model 2: AlzScore	-0.0±0.07, 0.95	0.01±0.09, 0.92	0.02±0.11, 0.83
Model 2: DASH	0.02±0.09, 0.81	-0.05±0.10, 0.65	0.20±0.19, 0.28
Model 2: AlzScore×DASH	0.01±0.05, 0.79	0.01±0.05, 0.82	-0.03±0.10, 0.79
Model 3: AlzScore	-0.0±0.07, 0.95	-0.0±0.09, 0.94	0.02±0.11, 0.85
Model 3: MAR	0.0±0.01, 0.77	0.0±0.0, 0.60	-0.0±0.02, 0.79

Model 3: AlzScore×MAR	0.0±0.0, 0.91	-0.0±0.0, 0.57	0.0±0.01, 0.64
California Verbal Learning Test, CVLT, List A			
Model 1: AlzScore	**-0.33*±0.17, 0.05**	**-0.42*±0.24, 0.08**	-0.19±0.27, 0.48
Model 1: HEI2010	-0.03±0.03, 0.22	-0.0±0.04, 0.93	**-0.08*±0.05, 0.09**
Model 1: AlzScore×HEI2010	0.01±0.02, 0.40	-0.01±0.02, 0.74	0.02±0.02, 0.34
Model 2: AlzScore	**-0.36**±0.17, 0.04**	**-0.42*±0.24, 0.08**	-0.27±0.28, 0.33
Model 2: DASH	-0.19±0.24, 0.43	-0.14±0.29, 0.63	-0.25±0.45, 0.58
Model 2: AlzScore×DASH	-0.10±0.13, 0.43	-0.02±0.16, 0.89	-0.31±0.24, 0.20
Model 3: AlzScore	**-0.34**±0.17, 0.05**	**-0.43*±0.25, 0.09**	-0.24±0.28, 0.40
Model 3: MAR	**-0.02*±0.02, 0.08**	-0.02±0.02, 0.34	**-0.09**±0.04, 0.03**
Model 3: AlzScore×MAR	0.0±0.0, 0.78	-0.0±0.01, 0.93	-0.0±0.02, 0.80
CVLT, Free Recall Long Delay (FRLD)			
Model 1: AlzScore	**-0.43***±0.17, 0.01**	**-0.48**±0.24, 0.05**	-0.36±0.27, 0.19
Model 1: HEI2010	-0.03±0.03, 0.28	0.0±0.04, 0.91	-0.08±0.05, 0.14
Model 1: AlzScore×HEI2010	0.01±0.02, 0.44	0.0±0.02, 0.97	0.02±0.03, 0.36
Model 2: AlzScore	**-0.46***±0.17, 0.009**	**-0.49**±0.24, 0.04**	-0.43±0.27, 0.12
Model 2: DASH	-0.24±0.24, 0.31	-0.13±0.29, 0.66	-0.50±0.45, 0.27
Model 2: AlzScore×DASH	-0.09±0.13, 0.48	-0.06±0.16, 0.71	-0.20±0.24, 0.41
Model 3: AlzScore	**-0.43***±0.17, 0.01**	**-0.50**±0.25, 0.05**	-0.45±0.28, 0.12
Model 3: MAR	**-0.03*±0.02, 0.09**	-0.01±0.02, 0.49	**-0.08**±0.04, 0.04**

Model 3: AlzScore×MAR	0.0±0.0, 0.65	-0.0±0.01, 0.74	0.0±0.02, 0.85
Benton Visual Retention Test, , BVRT			
Model 1: AlzScore	**0.37**±0.17, 0.03**	**0.67***±0.25, 0.007**	0.04±0.25, 0.88
Model 1: HEI2010	0.01±0.03, 0.84	-0.0±0.04, 0.97	0.04±0.05, 0.45
Model 1: AlzScore×HEI2010	0.0±0.01, 0.89	-0.01±0.02, 0.73	0.01±0.02, 0.63
Model 2: AlzScore	**0.37**±0.18, 0.04**	**0.68***±0.25, 0.008**	0.09±0.25, 0.73
Model 2: DASH	-0.19±0.25, 0.43	-0.28±0.30, 0.35	0.09±0.43. 0.84
Model 2: AlzScore×DASH	0.01±0.12, 0.92	-0.12±0.16, 0.45	0.27±0.23, 0.24
Model 3: AlzScore	**0.35**±0.18, 0.05**	**0.67***±0.26, 0.01**	-0.0±0.25, 0.98
Model 3: MAR	**-0.03**±0.02, 0.04**	**-0.03*±0.02, 0.08**	-0.01±0.04, 0.77
Model 3: AlzScore×MAR	-0.0±0.0, 0.73	0.0±0.01, 0.96	0.01±0.02, 0.45
Clock, Command			
Model 1: AlzScore	-0.02±0.04, 0.60	-0.04±0.06, 0.45	0.01±0.06, 0.89
Model 1: HEI2010	-0.0±0.0, 0.74	-0.1±0.01, 0.21	0.01±0.01, 0.32
Model 1: AlzScore×HEI2010	0.0±0.0, 0.10	0.01±0.0, 0.17	0.0±0.01, 0.46
Model 2: AlzScore	-0.02±0.04, 0.66	-0.03±0.05, 0.54	0.01±0.06, 0.86
Model 2: DASH	-0.05±0.05, 0.31	-0.08±0.07, 0.23	-0.01±0.09, 0.89
Model 2: AlzScore×DASH	0.02±0.03, 0.49	0.02±0.03, 0.57	0.01±0.05, 0.88
Model 3: AlzScore	-0.0±0.04, 0.82	0.0±0.06, 0.94	0.02±0.06, 0.70
Model 3: MAR	0.0±0.0, 0.16	0.0±0.0, 0.69	0.01±0.01, 0.18
Model 3: AlzScore×MAR	0.0±0.0, 0.13	**0.0**±0.0, 0.04**	-0.0±0.0, 0.38

Brief Test of Attention			
Model 1: AlzScore	-0.06±0.08, 0.45	-0.16±0.11, 0.14	0.11±0.11, 0.30
Model 1: HEI2010	-0.01±0.01, 0.24	-0.01±0.02, 0.37	-0.01±0.02, 0.77
Model 1: AlzScore×HEI2010	0.01±0.01, 0.22	0.0±0.01, 0.68	0.01±0.01, 0.29
Model 2: AlzScore	-0.06±0.08, 0.42	-0.17±0.11, 0.13	0.10±0.11, 0.40
Model 2: DASH	-0.07±0.10, 0.48	-0.09±0.13, 0.49	-0.09±0.19, 0.65
Model 2: AlzScore×DASH	-0.01±0.05, 0.91	-0.0±0.06, 0.95	-0.04±0.11, 0.68
Model 3: AlzScore	-0.04±0.08, 0.60	-0.11±0.11., 0.33	0.11±0.11, 0.31
Model 3: MAR	**0.01**±0.01, 0.04**	0.01±0.01, 0.15	**0.03*±0.02, 0.08**
Model 3: AlzScore×MAR	0.0±0.0, 0.22	0.01±0.01, 0.26	0.0±0.0, 0.88
Trailmaking Test, Part A			
Model 1: AlzScore	-2.86±1.80, 0.11	-3.59±2.19, 0.10	-4.76±3.12, 0.13
Model 1: HEI2010	-0.01±0.32, 0.98	-0.01±0.35, 0.98	-0.10±0.61, 0.87
Model 1: AlzScore×HEI2010	0.10±0.17, 0.51	0.12±0.19, 0.51	0.14±0.31, 0.64
Model 2: AlzScore	-2.62±1.79, 0.15	-3.37±2.17, 0.12	-4.06±3.08, 0.19
Model 2: DASH	-3.49±2.53, 0.17	-1.65±2.58, 0.53	-8.54±5.33, 0.11
Model 2: AlzScore×DASH	2.02±1.33, 0.13	1.62±1.36, 0.24	4.62±2.83, 0.11
Model 3: AlzScore	-2.90±1.81, 0.11	-3.63±2.31, 0.12	-5.08±3.14, 0.11
Model 3: MAR	-0.05±0.18, 0.78	-0.02±0.17, 0.91	0.04±0.51, 0.93
Model 3: AlzScore×MAR	-0.0±0.10, 0.93	-0.02±0.10, 0.82	0.05±0.23, 0.83
Trailmaking Test, Part B			
Model 1: AlzScore	-5.81±5.50, 0.29	0.84±8.44, 0.92	-14.23±7.72, 0.07

Model 1: HEI2010	-0.22±0.96, 0.82	-0.57±1.32, 0.67	1.83±1.50, 0.23
Model 1: AlzScore×HEI2010	-0.70±0.50, 0.17	-0.63±0.72, 0.34	-0.95±0.75, 0.21
Model 2: AlzScore	-6.37±5.52, 0.25	-0.16±8.32, 0.98	-12.53±7.82, 0.11
Model 2: DASH	7.94±7.79, 0.31	14.92±9.91, 0.13	-8.21±13.53, 0.55
Model 2: AlzScore×DASH	-4.85±4.09, 0.24	-6.94±5.20, 0.18	-0.51±7.20, 0.94
Model 3: AlzScore	-6.20±5.57, 0.27	-1.14±8.92, 0.90	-12.47±7.83, 0.11
Model 3: MAR	0.06±0.54, 0.92	-0.25±0.66, 0.71	1.08±1.26, 0.40
Model 3: AlzScore×MAR	-0.18±0.30, 0.55	-0.15±0.39, 0.70	0.09±0.57, 0.88
Digits Span, Forward			
Model 1: AlzScore	0.01±0.06, 0.80	-0.02±0.08, 0.82	0.11±0.09, 0.22
Model 1: HEI2010	**0.01*±0.01, 0.09**	0.01±0.01, 0.17	0.01±0.02, 0.49
Model 1: AlzScore×HEI2010	0.01±0.01, 0.33	-0.0±0.01, 0.93	0.01±0.01, 0.10
Model 2: AlzScore	0.02±0.06, 0.72	-0.02±0.08, 0.84	0.12±0.10, 0.20
Model 2: DASH	-0.07±0.08, 0.39	0.02±0.10, 0.84	-0.25±0.16, 0.11
Model 2: AlzScore×DASH	0.03±0.03, 0.50	0.01±0.05, 0.83	0.07±0.08, 0.40
Model 3: AlzScore	0.02±0.06, 0.77	-0.05±0.09, 0.60	0.10±0.10, 0.29
Model 3: MAR	0.01±0.01, 0.32	0.01±0.01, 0.23	0.0±0.01, 0.84
Model 3: AlzScore×MAR	-0.0±0.0, 0.85	-0.0±0.0, 0.34	0.0±0.0, 0.99
Digits Span, Backward			
Model 1: AlzScore	-0.03±0.05, 0.52	**-0.14*±0.07, 0.07**	0.08±0.08, 0.32
Model 1: HEI2010	-0.01±0.01, 0.21	-0.01±0.01, 0.43	-0.01±0.02, 0.58
Model 1: AlzScore×HEI2010	0.0±0.01, 0.91	0.0±0.01, 0.98	-0.0±0.01, 0.98

Model 2: AlzScore	-0.04±0.05, 0.47	**-0.14*±0.08, 0.06**	0.06±0.08, 0.48
Model 2: DASH	-0.07±0.08, 0.35	-0.14±0.09, 0.11	0.08±0.014, 0.55
Model 2: AlzScore×DASH	-0.02±0.04, 0.56	-0.01±0.05, 0.89	-0.10±0.08, 0.20
Model 3: AlzScore	-0.04±0.06, 0.51	**-0.17**±0.08, 0.04**	0.09±0.08, 0.28
Model 3: MAR	0.0±0.01, 0.55	0.0±0.01, 0.25	0.0±0.01, 0.98
Model 3: AlzScore×MAR	-0.0±0.0, 0.84	-0.0±0.0, 0.29	-0.0±0.0, 0.53
Card Rotation test			
Model 1: AlzScore	0.14±0.56, 0.80	0.40±0.81, 0.62	-0.90±0.85, 0.29
Model 1: HEI2010	-0.07±0.09, 0.44	-0.08±0.12, 0.52	0.17±0.18, 0.93
Model 1: AlzScore×HEI2010	**0.11**±0.05, 0.04**	0.10±0.07, 0.13	0.08±0.10, 0.44
Model 2: AlzScore	0.14±0.56, 0.80	0.50±0.81, 0.54	-1.20±0.85, 0.16
Model 2: DASH	0.11±0.77, 0.89	-0.24±0.95, 0.80	1.65±1.41, 0.25
Model 2: AlzScore×DASH	0.45±0.44, 0.31	0.73±0.54, 0.18	-0.59±0.83, 0.47
Model 3: AlzScore	0.06±0.56, 0.91	0.31±0.84, 0.72	-1.07±0.84, 0.21
Model 3: MAR	0.06±0.05, 0.28	0.03±0.06, 0.60	0.20±0.15, 0.19
Model 3: AlzScore×MAR	-0.02±0.03, 0.56	-0.02±0.04, 0.63	0.03±0.07, 0.66
Identical Pictures			
Model 1: AlzScore	0.23±0.18, 0.22	0.25±0.27, 0.35	0.18±0.28, 0.53
Model 1: HEI2010	-0.03±0.03, 0.32	-0.04±0.04, 0.35	-0.02±0.06, 0.72
Model 1: AlzScore×HEI2010	**0.03*±0.02, 0.07**	0.02±0.02, 0.31	0.05±0.03, 0.13
Model 2: AlzScore	0.22±0.19, 0.24	0.27±0.27, 0.32	0.10±0.29, 0.74
Model 2: DASH	0.02±0.26, 0.94	0.02±0.31, 0.94	0.07±0.49, 0.88

Model 2: AlzScore×DASH	0.14±0.15, 0.35	0.25±0.18, 0.16	-0.10±0.28, 0.71
Model 3: AlzScore	0.20±0.19, 0.28	0.25±0.28, 0.38	0.05±0.29, 0.87
Model 3: MAR	-0.01±0.02, 0.54	-0.02±0.02, 0.30	0.08±0.05, 0.15
Model 3: AlzScore×MAR	0.0±0.01, 0.74	0.0±0.01, 0.85	0.02±0.02, 0.39
Animal Fluency			
Model 1: AlzScore	0.05±0.14, 0.72	-0.17±0.19, 0.39	0.29±0.24, 0.24
Model 1: HEI2010	-0.01±0.03, 0.68	-0.02±0.03, 0.56	-0.01±0.05, 0.82
Model 1: AlzScore×HEI2010	**0.03**±0.01, 0.02**	**0.04**±0.02, 0.02**	0.02±0.02, 0.37
Model 2: AlzScore	0.08±0.15, 0.58	-0.10±0.19, 0.59	0.26±0.25, 0.29
Model 2: DASH	-0.11±0.21, 0.60	-0.16±0.23, 0.47	0.02±0.42, 0.96
Model 2: AlzScore×DASH	**0.18*±0.11, 0.09**	**0.25**±0.12, 0.04**	-0.01±0.22, 0.98
Model 3: AlzScore	0.08±0.15, 0.60	-0.07±0.20, 0.72	0.25±0.24, 0.30
Model 3: MAR	0.01±0.01, 0.38	0.01±0.02, 0.56	0.03±0.04, 0.51
Model 3: AlzScore×MAR	0.0±0.0, 0.32	0.01±0.01, 0.51	0.01±0.02, 0.71

Abbreviations: AlzScore= Alzheimer's Risk Score; MMSE= Mini-Mental State Examination; CVLT-List A= California Verbal Learning test- List A; CVLT-DFR= California Verbal Learning Test-Delayed Free Recall; BVRT= Benton Visual Retention Test; Attention= Brief Test of Attention; Trails A= Trailmaking Test A; Trails B= Trailmaking Test B; Digit Span Forward= Digits Span Forward Test; Digit Span Backward= Digits Span Backward Test; Clock Command= Clock Command Test; Identical Pictures= Identical Pictures Test; Card Rotation= Card rotation Test; Animal Fluency= Animal Fluency Test. *** p<0.01, ** p<0.05, * p<0.10 (numbers are highlighted according to these cutoffs. Might change based on what you think should be the best to avoid Type I errors).

1 OLS regression models, for men and women combined and stratified by sex were adjusted for age, sex, race, poverty status, education status, BMI, total energy intake, current smoking status, current drug use, depression, Diabetes, Hypertension, Dyslipidemia, Cardiovascular Disease, Inflammatory conditions and use of Non-Steroidal Anti-Inflammatory Drugs, NSAIDs. Covariates were centered at the mean. 2-way interaction terms were added for AlzScore and dietary quality indices. Dietary quality indices for Models 1-3 were HEI-2010, DASH and MAR, respectively. Main effects of those exposures were included along with main effects of covariates. ample sizes for each model and stratum can be found in **Table 2.**

Table S4. (b): Two-way interaction (p-values) of cognitive performance test scores, select SNP[1§]s and DASH[2] by sex at baseline, for HANDLS participants ≥ 50y of age.

Cognitive Tests/ SNPs	**Women**	**Men**
Benton Visual Retention Test (BVRT)		
rs165932_T	-	2.34±1.09**, 0.03
rs1799990_A	-1.13±0.60*, 0.06	-
rs2251101_T*	2.05±1.22*, 0.09	-
rs2306604_C	2.13±1.10**, 0.05	-
Clock, Command		
rs1049296_C	-0.89±0.32***, 0.006	-
rs165932_T	-0.36±0.18**, 0.04	-
rs449647_A	-0.44±0.18**, 0.02	-
Brief Test of Attention		
rs1049296_C	-1.16±0.62*, 0.06	-
rs165932_T	-0.74±0.33**, 0.03	-
Trailmaking Test, Part A		
rs1049296_C*	-	-65.18±21.94***, 0.004
rs4291_A	-9.27±5.41*, 0.09	-
rs4806173_C*	-	-36.07±13.46***, 0.008
Trailmaking Test, Part B		
rs1049296_C	112.89±49.42**, 0.02	-
rs165932_T	52.12±27.41*, 0.06	-

rs2306604_C	83.42±36.23**, 0.02	-
rs4291_A	38.13±20.98*, 0.07	-
rs4343_A	45.88±26.18*, 0.08	-
Digits Span, Forward		
rs1064039_A	-	-0.52±0.22**, 0.02
rs1799990_A	-	-0.79±0.42*, 0.06
Digits Span, Backward		
rs165932_T	-0.59±0.25**, 0.02	-
rs2251101_T	-	0.92±0.47*, 0.06
rs2306604_C	-0.69±0.33**, 0.04	-
rs4343_A*	-0.54±0.24**, 0.03	0.96±0.42**, 0.02
Card Rotation test		
rs1799990_A	-	-6.52±3.46*, 0.06
rs4343_A*	-	7.97±4.37*, 0.07
rs449647_A	-4.87±2.86*, 0.09	-
rs4806173_C*	-	9.07±3.41***, 0.009
Identical Pictures		
rs4343_A*	-	3.18±1.46**, 0.03
Animal Fluency		
rs2251101_T	-1.64±0.92*, 0.08	-
rs4806173_C	1.12±0.61*, 0.07	-
rs4845378_G*	4.76±1.07***, 0.000	-

[1] rs##_allele refers to the selected SNP dosages that were incorporated into the AlzScore. The SNP dosage is coded as: 0: no allele, 1: 1 allele, 2: 2 alleles. [2] Tests with no significant three-way interactions with DASH: Mini- Mental State Examination (MMSE); California Verbal Learning Test (CVLT), List A; California Verbal Learning Test (CVLT), Free Recall Long Delay (FRLD)*** p<0.01, ** p<0.05, * p<0.1. *Also presented with statistically significant (p<0.10) three-way interactions: Diet X SNP X Sex. [§] This SNP dosage (unlike in this Table) was reverse coded to create the AlzScore, since the alternative allele was shown to increase the risk of adverse cognitive outcomes,including Alzheimer's Disease.

Table S4. (c): Two-way interaction (p-values) of cognitive performance test scores, select SNP[1§]s and MAR[2] by sex at baseline, for HANDLS participants ≥ 50y of age.

Cognitive Tests/ SNPs	**Women**	**Men**
Mini- Mental State Examination (MMSE), total score		
rs2306604_C*	0.04±0.02**, 0.02	-
California Verbal Learning Test (CVLT), List A		
rs1799990_A	0.07±0.04*, 0.09	-
California Verbal Learning Test (CVLT), Free Recall Long Delay (FRLD)		
rs1799990_A	0.07±0.04*, 0.09	-
Benton Visual Retention Test (BVRT)		
rs1049296_C*	-	-0.22±0.13*, 0.07
rs1064039_A	-0.04±0.02**, 0.05	-
rs2251101_T	-0.24±0.09***, 0.009	-
rs4806173_C	-0.14±0.08**, 0.05	-
Clock, Command		

rs1049296_C	-	0.06±0.03**, 0.02
rs165932_T	0.02±0.01**, 0.02	-
rs4291_A*	-	0.03±0.02**, 0.03
rs449647_A*	0.02±0.01***, 0.006	-
rs4806173_C	0.48±0.12***, 0.003	-
Brief Test of Attention		
rs165932_T	0.06±0.02***, 0.004	0.07±0.03**, 0.04
rs4291_A	-	0.06±0.03*, 0.09
rs4806173_C	0.06±0.03**, 0.03	-
rs4845378_G	0.13±0.05**, 0.02	-
Trailmaking Test, Part B		
rs1049296_C*	-13.85±4.39***, 0.002	-
rs4845378_G	-7.29±4.11*, 0.08	-
Digits Span, Forward		
rs405509_A	0.02±0.01*, 0.08	-
rs4343_A	0.05±0.02*, 0.06	-
rs4806173_C*	0.05±0.03*, 0.09	-
Digits Span, Backward		
rs1799990_A*	s0.03±0.01**, 0.05	-
rs4343_A	0.04±0.02*, 0.06	-
Card Rotation test		
rs2251101_T*	0.44±0.26*, 0.09	1.61±0.46***, 0.001

rs2306604_C	-	0.73±0.31**, 0.02
rs4806173_C	-	0.65±0.28**, 0.02
Identical Pictures		
rs2306604_C	-	0.25±0.11**, 0.02
rs449647_A*	-	0.22±0.09**, 0.02
rs4806173_C	-	0.20± 0.10**, 0.04
Animal Fluency		
rs4845378_G	-	0.21±0.10**, 0.04

[1] rs##_allele refers to the selected SNP dosages that were incorporated into the AlzScore. The SNP dosage is coded as: 0: no allele, 1: 1 allele, 2: 2 alleles. [2] Tests with no significant three-way interactions with MAR: Trailmaking Test, Part A. *** p<0.01, ** p<0.05, * p<0.1.*Also presented with statistically significant (p<0.10) three-way interactions: Diet X SNP X Sex.[§] This SNP dosage (unlike in this Table) was reverse coded to create the AlzScore, since the alternative allele was shown to increase the risk of adverse cognitive outcomes, including Alzheimer's Disease.

Table S4. (d): Two-way interaction (p-values) of cognitive performance test scores, select SNP[1§]s and HEI-2010 by sex at baseline, for HANDLS participants ≥ 50y of age.

Cognitive Tests/ SNPs	**Women**	**Men**
Mini- Mental State Examination (MMSE), total score		
rs165932_T	-	-0.09±0.05*, 0.07
rs1799990_A*	-	0.09±0.05,* 0.07
rs4806173_C	0.05±0.03*, 0.09	-
California Verbal Learning Test (CVLT), List A		
rs1049296_C	0.30±0.20*, 0.07	-
rs4343_A	-	-0.28±0.16,* 0.08

California Verbal Learning Test (CVLT), Free Recall Long Delay (FRLD)		
rs1049296_C	0.31±0.16**, 0.05	-
rs4343_A	-	-0.27±0.16*, 0.09
Benton Visual Retention Test (BVRT)		
rs1799990_A	-	0.20±0.11*, 0.07
Clock, Command		
rs1049296_C*	-0.08±0.04**, 0.04	0.21±0.08***, 0.006
rs2251101_T	-	0.09±0.04**, 0.05
rs2306604_C	-0.04±0.03*, 0.09	-
rs4806173_C	0.03±0.02**, 0.05	-
Brief Test of Attention		
rs4806173_C	-	0.08±0.04*, 0.08
Trailmaking Test, Part A		
rs1049296_C*	-	-13.50±3.89***, 0.001
Trailmaking Test, Part B		
rs1049296_C*	-	-25.83±9.90**, 0.01
rs1064039_A	-	3.64±1.80**, 0.05
rs2306604_C	-	11.55±4.85**, 0.02
rs405509_A	-5.68±2.93**, 0.05	-
Digits Span, Forward		
rs4806173_C	-	0.11±0.04***, 0.008

Digits Span, Backward		
rs4343_A	-	0.08±0.05*, 0.09
Card Rotation test		
rs2306604_C*	-0.79±0.42*, 0.07	-
rs4806173_C	-	0.71±0.38*, 0.06
Identical Pictures		
rs1049296_C*	-	1.45±0.80*, 0.08
rs2306604_C*	-	0.48±0.18**, 0.01
rs4806173_C*	-	0.24±0.13*, 0.07
Animal Fluency		
rs4806173_C	0.14±0.07*, 0.06	0.24±0.10**, 0.03
rs4845378_G	0.33±0.15**, 0.02	-

[1] rs##_allele refers to the selected SNP dosages that were incorporated into the AlzScore. The SNP dosage is coded as: 0: no allele, 1: 1 allele, 2: 2 alleles. *Also presented with statistically significant (p<0.10) three-way interactions: Diet X SNP X Sex [§] This SNP dosage (unlike in this Table) was reverse coded to create the AlzScore, since the alternative allele was shown to increase the risk of adverse cognitive outcomes, including Alzheimer's Disease. Note: *** p<0.01, ** p<0.05, * p<0.1(will change based on your final cutoff and some results will not be significant anymore, e.g., MMSE rs_4806173C)

Supplementary material 1: Cognitive Tests

Mental Status – The Mini Mental State Examination (MMSE) concentrates only on the cognitive aspects of mental functions. It has eleven questions, easy to administer and is practical to use serially and routinely [1]. The MMS is divided into two sections. First section requires vocal responses only and covers orientation, memory, and attention; with a maximum score of 21. Second section tests the ability to name, follow verbal and written commands, with a maximum score is nine. Because of the reading and writing involved in Part II, patients with severely impaired vision may have some extra difficulty that can usually be eased by large writing and allowed for in the scoring.

Verbal learning and memory—The California Verbal Learning Test-II (CVLT-II) [2] as described by Delis *et al.* measures verbal learning and memory by immediate and delayed recall and recognition of two sixteen-word lists. As administered to HANDLS participants, List A consists of three trials, followed by list B with one trial. After testing with list B, short-delay short free recall and cued recall of list A are tested. After ~ 30 min delay, long-delay free recall, cued recall and yes/no-recognition attempts of list A conclude the test.

Non-verbal memory—The Benton Visual Retention Test [3] consists of ten designs and their reproduction by participants, as accurately as possible. It measures visual memory, perception and visuo-constructional abilities. It is an untimed test scored by the examiner with a reliability range of 0·74 to 0·84 [4].

Working memory—The Digit Span subscale of the Wechsler Adult Intelligence Scale consists of forward and backward test measurements [5]. In both tests, seven pairs of increasing, random number sequences, are presented verbally at a rate of one per second. In Digits Forward, the subject repeats the same number sequence after the examiner. In Digits Backward the participant is asked to repeat the number sequence in reverse order. The test is discontinued when the subject fails both the forward and backward trial of any given sequence string. Digits Forward and Backward are measured by the number of correct trials. This is used as a valid test of attention, working memory and concentration.

Attention and cognitive flexibility—The Trail Making Test [6] is administered in two timed-parts, lasting 5 to 10 min each. Trails A is administered first, where subjects connect, in ascending order, randomly numbered circles on a page by drawing lines. In Trails B, subjects connect alternating numbered and lettered circles, in ascending order the same way as Trails A. Following of the correct sequence is required to complete each test. Cognitive task burden in Trails B is greater than Trails A. The Trails Making Test, therefore, provides a composite measure of attention, visuomotor tracking, and cognitive flexibility.

Visuospatial ability—The Card Rotations Test measures two-dimension visuospatial ability with the help of different card shapes [7]. Ten rows of eight cards each are compared with a sample card shape to determine if each card is rotated or flipped over. The completion is marked by identifying two sets of ten card rows in 3 min each. The score is the difference between cards marked correctly vs. incorrectly.

Perceptual speed—The Identical Pictures Test includes three components of perceptual speed: perceptual fluency, decision-making speed and immediate perceptual memory. In this timed test, sample objects are matched with an identical picture in the adjacent row of test objects [7]. The score is the number of correct answers, minus a fraction of the number of incorrect answers.

Verbal fluency—The Verbal Fluency Test is used to assess spontaneous generation of words from specific categories in a preset amount of time [4]. In HANDLS, participants were asked to name as many animals as possible within 60s. Then, the total number of unique animals named is aggregated to generate a categorical animal fluency score.

1. Folstein Mf Fau - Folstein SE, Folstein Se Fau - McHugh PR, McHugh PR. "Mini-mental state". A practical method for grading the cognitive state of patients for the clinician.
2. Delis D KJ, Kaplan E et al. CVLT-II. New York: The Psychological Corporation. 2000.
3. AL B. Benton Visual Retention Test, 5th ed. San Antonio, TX: The Psychological Corporation. 1992.

4. Strauss E SESO. A Compendium of Neuropsychological Tests: Administration, Norms and Commentary, 3rd ed. New York: Oxford University Press. 2006.
5. D W. Wechsler Adult Intelligence Scale – Revised. New York: The Psychological Corporation. 1981.
6. RM R. Trail Making Test: Manual for administration and scoring. Tucson, AZ: Reitan Neuropsychology Laboratory. 1992.
7. Ekstrom RB FJ, Harman HH et al. . Kit of Factor-Referenced Cognitive Tests, revised ed. Princeton, NJ: Educational Testing Service. 1976.

Supplementary material 2: **Diet Quality Interaction**

Upon correction for multiple testing ($P<0.0028$), HEI-2010 was linked to better performance on Trails B test (i.e. lower score) among men with higher dosage of the rs1049296 SNP ("C" allele, entered as is in the hAlzScore), indicating a synergistic interaction between diet quality using HEI-2010 and AD genetic risk for performance on this test of executive function (**Table S3a**). In contrast, among women, DASH was linked to better performance on a test of Animal Fluency with higher dosage of the rs4845378 ("G" allele, entered inverted in the hAlzScore), indicating an antagonistic interaction between diet quality AD genetic risk for performance on this test of verbal fluency. This interaction differed significantly between men and women ($P<0.10$, 3-way interaction between SNP, DASH and sex) (**Table S3b**). Based on models with MAR score interacting with individual SNPs (**Table S3c**), among women, MAR was linked to a better performance on Trails B (i.e. lower score), with higher "C" allele dosage on rs1049296 (SNP entered as is in hAlzScore). This indicates that there was a synergistic interaction between poor diet quality using MAR and AD genetic risk in the case of Trails B among women. Finally, among men, MAR was associated with better performance on card rotation for individuals with greater "T" allele dosage on rs2251101, indicating a synergistic interaction between poor diet quality as measured by MAR and AD genetic risk in men for this test of visuospatial ability. Both latter interactions differed significantly between men and women ($P<0.10$, 3-way interaction between SNP, MAR and sex).

Supplementary material 3: Power Calculation

We used STATA for all our main analyses and R(3.6.2) for power calculation. We then used STATA to verify the observed findings from R.

For Animal Fluency and hAlzScore interactions with HEI2010 (overall) :

```
      Source |       SS           df       MS      Number of obs   =       307
-------------+----------------------------------   F(21, 285)      =      2.43
       Model |  1095.84227        21  52.1829654   Prob > F        =    0.0006
    Residual |  6113.37597       285   21.450442   R-squared       =    0.1520
-------------+----------------------------------   Adj R-squared   =    0.0895
```

Total | 7209.21824 306 23.5595367 Root MSE = 4.6315

To calculate power for this association, we used the function ”pwr” in R (https://www.statmethods.net/stats/power.html).

This model uses degrees of freedom (u= numerator, and v=denominator) with designated effect sizes as recommended by the author (f2=0.02 is for small, f2=0.15 is for medium and f2=0.35 I for large effects). We present the power of our associations for all three levels (at p=0.05) below:

```
> pwr.f2.test(u = 21, v = 306, f2 = 0.02, sig.level = 0.05, power =NULL )
    Multiple regression power calculation
             u = 21
             v = 306
            f2 = 0.02
     sig.level = 0.05
         power = 0.2353091
> pwr.f2.test(u = 21, v = 306, f2 = 0.15, sig.level = 0.05, power =NULL )
Multiple regression power calculation
             u = 21
             v = 306
            f2 = 0.15
     sig.level = 0.05
         power = 0.9967409
> pwr.f2.test(u = 21, v = 306, f2 = 0.35, sig.level = 0.05, power =NULL )
    Multiple regression power calculation
  u = 21
             v = 306
            f2 = 0.35
     sig.level = 0.05
         power = 1
```

For residuals,

```
> pwr.f2.test(u = 18, v = 285, f2 = 0.35, sig.level = 0.05, power =NULL )
     Multiple regression power calculation
                    u = 21
              v = 285
             f2 = 0.35
      sig.level = 0.05
          power = 1
```

For Animal Fluency and hAlzScore interactions with HEI2010 (women) :

```
      Source |       SS           df       MS              Number of obs   =       162
-------------+----------------------------------        F(20, 141)      =      1.39
       Model |   495.74861        20  24.7874305   Prob > F        =    0.1382
    Residual |   2519.8625       141  17.8713653   R-squared       =    0.1644
-------------+----------------------------------      Adj R-squared   =    0.0459
       Total |  3015.61111       161  18.7305038     Root MSE        =    4.2275
```

```
> pwr.f2.test(u = 21, v = 161, f2 = 0.02, sig.level = 0.05, power =NULL )
Multiple regression power calculation
              u = 21
              v = 161
             f2 = 0.02
      sig.level = 0.05
          power = 0.1327356
> pwr.f2.test(u = 21, v = 161, f2 = 0.15, sig.level = 0.05, power =NULL )
     Multiple regression power calculation
              u = 21
              v = 161
```

f2 = 0.15

sig.level = 0.05

power = 0.8735838

> pwr.f2.test(u = 21, v = 161, f2 = 0.35, sig.level = 0.05, power =NULL)

Multiple regression power calculation

u = 21

v = 161

f2 = 0.35

sig.level = 0.05

power = 0.9996864

For Animal Fluency:

We used STATA to verify the calculations using “powersim” command with similar effect sizes (0.1, 0.2 and 0.3). **For main effects:**

Power analysis simulations

Effect sizes b: .1 .2 .3

H0: b = 0

Sample sizes: 100 120 140 160 180 200 220 240 260 280 300

alpha: .05

N of replications*: 500

do-file used for data generation: psim_dofile

Model command: reg y x1 x2

Power by sample and effect sizes:

| Specified effect

Sample | size

size	.1	.2	.3
100	0.146	0.352	0.742
120	0.148	0.466	0.856
140	0.172	0.516	0.868
160	0.186	0.566	0.892
180	0.214	0.646	0.928
200	0.202	0.674	0.974
220	0.274	0.764	0.970
240	0.282	0.760	0.984
260	0.248	0.806	0.994
280	0.344	0.824	0.992
300	0.294	0.828	0.992

Total N of requested MC replications: 16500

Total N of successful MC replications: 16500

* per sample and effect size combination

For Interactions:

Power analysis simulations

Effect sizes b: .1 .2 .3

H0: b = 0

Sample sizes: 100 120 140 160 180 200 220 240 260 280 300

alpha: .05

N of replications*: 500

do-file used for data generation: psim_dofile2

Model command: reg y c.x1##c.x2

Power by sample and effect sizes:

Sample size	Specified effect size .1	.2	.3
100	0.132	0.362	0.692
120	0.124	0.428	0.816
140	0.174	0.482	0.864
160	0.156	0.564	0.894
180	0.208	0.634	0.932
200	0.234	0.650	0.940
220	0.274	0.724	0.970
240	0.246	0.780	0.970
260	0.306	0.786	0.992
280	0.276	0.840	0.988
300	0.308	0.862	0.996

Total N of requested MC replications: 16500

Total N of successful MC replications: 16500

* per sample and effect size combination

Our estimates from two different statistical software show that they are comparable, and the exact numbers are given above.
